# Testing for the footprints of stabilization economic policy in forecast errors

**DOI:** 10.1371/journal.pone.0336495

**Published:** 2025-12-01

**Authors:** Wojciech Charemza, Christian Francq, Radu Lupu, Svetlana Makarova, Jean-Michel Zakoïan

**Affiliations:** 1 Vistula University, Poland; 2 University of Leicester, United Kingdom; 3 CREST, Paris, France; 4 Bucharest University of Economic Studies and Institute for Economic Forecasting, Romanian Academy, Romania; 5 University College London, United Kingdom; Università Cattolica del Sacro Cuore Sede di Piacenza e Cremona Facoltà di Economia: Universita Cattolica del Sacro Cuore Facolta di Economia e Giurisprudenza, ITALY

## Abstract

This paper introduces a novel statistical test, the Policy Effects Lagrange Multiplier (PELM) test, to detect stabilization policy effects in the distribution of forecast errors from dynamic financial models. Traditional analyses of policy impact typically rely on explicit policy information or direct intervention data, which are often unavailable or incomplete. In contrast, the proposed PELM test infers policy footprints from the distribution of forecast errors alone. Empirically applied to sovereign bond yield data from 33 countries before the Russian financial crisis of 2014, the test identifies countries showing stabilization policy footprints. Subsequent analysis shows that significant budgetary improvements were observed for years following the crisis in the group of countries where our test statistically confirmed stabilization policies. This confirms the rationale of test foundations and also indicates its predictive properties. Robustness checks further validate these findings across various model specifications and sensitivity scenarios. The proposed PELM test offers policymakers and researchers a powerful tool for evaluating stabilization policies, facilitating better forecasting and assessing policy efficiency in diverse economic contexts without necessitating detailed policy intervention data.

## 1 Introduction

This paper has two primary aims. The first aim is to propose a test for identifying stabilization policy effects (or ‘footprints’) in the distribution of forecast errors generated by a dynamic financial market model with time-varying volatility of a policy-dependent variable. The second aim is to demonstrate how the results of this test can effectively enhance the prediction of fiscal indicator dynamics.

The commonly used approaches to testing policy outcomes mainly focus on analyzing how the policy impacts the conditional mean of the modeled process. These methods typically involve identifying policy effects by using explicit information about the timing or magnitude of interventions and then applying econometric tools such as structural factor analysis, regime-switching techniques, or textual measures of policy communication (see, for example, Basistha and Kurov [1]; Forni and Gambetti [2]; Hanish [3]; Klingelhöfer and Sun [4]; Guidolin and Pedio [5]). Studies like Ehrmann and Fratzscher [6]; Nakamura and Steinsson [7]; and especially Hoesch, Rossi, and Sekhposyan [8], expanded this framework by examining how both information advantage and information channels evolve over time, demonstrating that the effectiveness of policy communication differs across various economic environments and time periods.

Therefore, if one considers the effects of policy decisions on higher moments of market indicators, most notably the variance of ex-ante forecast errors, usually associated with a measure of forecast uncertainty, the problem of data for identifying the factors affecting these becomes more intricate. From a theoretical perspective, the difficulty of identifying the timing and nature of events that reduce forecast uncertainty arises because uncertainty itself, if understood in the Knight [9] sense, is not directly observable. It can, however, be inferred from the distribution of forecast errors (see Rossi, Sekhposyan and Soupre [10]). In the rational expectations framework, information asymmetries imply that policy interventions can affect uncertainty through two channels: by altering the information set available to agents, thereby narrowing the dispersion of beliefs (see Lucas [11]), or by influencing second-order expectations, that is, beliefs about the beliefs of others (see Morris and Shin [12]). In both cases, the effect manifests not in the conditional mean of forecasts but in the higher moments of their distribution, particularly variance. This creates an identification challenge: variance reductions may stem from structural shocks directly targeting uncertainty, such as new regulatory frameworks or systematic changes in central bank communication, or other factors that incidentally dampen volatility. Hence, while the theoretical literature emphasizes the role of credible policy, transparency, and communication in stabilizing expectations, empirical analysis must grapple with the difficulty of mapping such abstract channels onto specific, datable events. In practice, there is often a plethora of such factors that are difficult to quantify or even date, but undoubtedly have some effects on the stabilization of financial markets. This information might either (a) represent direct decisions leading to reduced uncertainty or (b) be the indirect cause of such decisions.

Below are two rather evident examples of (a) above: decisions to stabilize the government bonds market. The first example is the MiFID II (Markets in Financial Instruments Directive II) Implementation Plans by the European Commission. It reduced uncertainty premiums while the regulatory framework remained ambiguous, particularly in corporate bond markets where transparency requirements were changing significantly (see, e.g., Kaya [13]). However, its timing is not clearly identified. It was announced on January 15, 2014 (political agreement reached); final technical standards were published on April 15, 2016. Another example is the evolution of communication between 2001 and 2005 at the European Central Bank (ECB). It occurred through a series of gradual changes to the Bank’s communication practices. In late 2001, the ECB began using specific signaling phrases in its statements. Throughout 2002–2003, it standardized its communication patterns, and by 2004–2005, it had established a predictable format for including forward-looking qualitative projections in its statements. The entire process stabilized European bond markets, resulting in a gradual decline in forecast errors for ECB policy decisions and corresponding reductions in yield volatility around ECB announcements (see, e.g., Jansen and De Haan [14]). Many more examples can be given that are related to different countries and markets.

Regarding (b), it is much more challenging to come up with examples. It may be real-time information from experts, politicians, and independent forecasters, which serves as an early warning, creating a stabilizing effect. Sometimes, such information can be identified; during the 2008 financial crisis, Warren Buffett published the op-ed entitled ‘Buy American. I Am’ in the *New York Times* (October 16, 2008) significantly helped to stabilize markets by expressing long-term confidence when panic was widespread. However, this might be very difficult in most cases due to the diversified and sometimes confidential or secret nature of this information.

How do we deal with all this information coming at different times, interacting with each other, and generally not known to the researcher? They surely have some effects on forecast uncertainty, some stabilizing it, and some do not. We propose a sort of reverse engineering approach here. We focus on identifying policy outcomes rather than explicitly tracing the policy interventions themselves. It builds on the findings of Charemza, Díaz, and Makarova [15], who showed that, under reasonable assumptions, the empirical distribution of ex-ante forecast errors from an econometric model of policy-dependent economic indicators can be approximated by the weighted skew-normal (WSN) distribution. The parameters of this distribution can be interpreted as outcomes of actions based on qualitative and quantitative signals received from various sources, such as banks’ statements, communications from independent forecasters, and economic commentators. However, estimating these parameters is challenging due to numerical difficulties and restrictions that must be imposed on the parameters. The test proposed in this paper, which is based on the Lagrange Multiplier principle and is called the PELM test (the Policy Effects Lagrange Multiplier test) throughout this paper, can be used for detecting footprints of economic policy in forecast errors without the need for such estimation.

In other words, we can test for the effects of non-econometric forecasting, e.g., by experts, professional forecasters, influencers, gurus, or economic analysts in cases where such forecasts’ data are unavailable. In fact, except for a few countries, such data are not available or do not exist in an explicit form, as experts’ opinions and advice are often presented not transparently. Alternatively, they might be produced in a fuzzy or indirect form and published in various media.

The quadratic loss function is a natural choice of the objective function in this context because reductions in forecast error variance directly lead to measurable improvements in predictive accuracy. At the same time, we recognize that alternative uncertainty measures have been suggested, including those based on asset-pricing models or non-quadratic loss frameworks (see, for example, David and Veronesi [16]). Our aim is not to dismiss these methods but to provide a complementary, testable mechanism: one that links stabilization policies to changes in the distribution of forecast errors without requiring detailed information about the timing or magnitude of policies.

The significance of the PELM test statistics is, evidently, only the necessary but not sufficient condition for claiming that the test proposed here indeed discovers footprints of a stabilization policy. As is often the case in abductive reasoning, there might be potentially different causes of such a result. This is why we conduct our research in two stages, which correspond to the two aims of this paper. In the first stage, we apply the PELM test to the ex-post forecast errors (forecast uncertainty) of a dynamic volatility model of government bond yields to detect possible policy intervention footprints. Then, in the second stage, we test whether significant budgetary changes confirm the stabilization effect.

Reduction in forecast uncertainty might also positively affect budget deficit through lower borrowing costs, increasing fiscal policy confidence, stimulating economic growth, and allowing more flexibility in central bank policy coordination (for evidence in developed markets, see, e.g., Peppel-Srebrny [17]; and, in particular, Jiang and Li [18], for comprehensive empirical evidence; for a broader context, see Manzli et al [19]).

Our empirical analysis focuses on finding traces of stabilization policy in the time series of sovereign bond yields for 33 countries. First, we apply the PELM test to forecast errors of a dynamic volatility model fitted to daily data on these yields. On the basis of such testing, we divide these countries into two groups: one where the test statistics are significant and the other containing the remaining countries. Concentrating on the aftermath of the Russian financial crisis in December 2014, in the second stage of testing, we apply the paired t-test separately for both groups to the difference in budget deficits in the first year of the crisis and, subsequently, two, three, and four years later, when the stabilization policy effects should be evident. The paired test statistic turns out to be significantly positive for the group of countries where footprints of stabilization policy have been confirmed, but not for the other group. In our view, this confirms the rationale of our testing approach and fulfils the second aim of this paper.

The remainder of the paper is organized as follows: in Section 2, we formalize the research problem and illustrate the rationale of our settings. Section 3 sets out the general assumptions underpinning our testing approach. It also describes two variants of our PELM test; one is computationally easier and has a chi-squared asymptotic distribution, but is based on rather strict assumptions. The other variant is computationally heavy but can be conducted under less restrictive assumptions. This section gives a detailed discussion of the primary asymptotic characteristics of both variants. Section 4 analyzes the practical aspects and properties of these tests in finite samples, particularly the use of simulated critical values and p-values. It also presents some results illustrating power of the tests. Section 5 addresses the second aim of the paper. This section discusses the paired test results for budget deficits separately for the countries with significant and non-significant PELM statistics. Section 6 summarizes the outcomes of various robustness checks. In particular, it demonstrates that our test can be of practical use for analyzing other global financial crises, such as the crisis caused by the unexpected outcome of the UK’s Brexit referendum in 2016. This empirical evidence confirms that the significance of the PELM statistics corresponds to creating positive fiscal effects. Section 7 concludes, highlighting broader potential applications and directions for future research. The paper is completed with Supplement 1 with generalizations, proofs of lemmas and propositions; Supplement 2 with additional simulation and empirical results; and Supplement 3 with the data used for empirical example and robustness analysis. The data is also available at data repository *figshare* at https://figshare.com/articles/dataset/Data_for_Testing_Footprints_xlsx/30016453?file=57852376.

### 2 Set-up and the testing problem

The problem of detecting the effects of policy interventions on the distribution of forecast errors can be formulated in the following way.

Let $m_{t}$ and $\sigma_{t}^{2}$ be the core predictions of the level and variance of an economic or financial variable $X_{t}$. These predictions rely entirely on information available at time t−1. This information is denoted by ${ℱ}_{t-1}$, and comprises the historical data of the economic variable, i.e., the values of $X_{u}$ for u<t. Thus, in the absence of economic policy intervention, the variable would be


$$X_{t}^{*}=m_{t}+\sigma_{t}U_{t}^{*},$$

with an error term $U_{t}^{*}$, which is assumed to be identically and independently distributed, *iid*, 𝒩(0,1). Next, assume that policymakers have access to a vector $s_{t}$ of additional information that includes news, information from policy advisors, or even rumors created by media influencers that arise after the core prediction was made. If such information had been publicly available prior to the time t it would have been included in ${ℱ}_{t-1}$ and thus in the core prediction. Let ${𝒮}_{t}$ be the sigma-field generated by $s_{t}-E(s_{t}|{ℱ}_{t-1})$, that is the part of the information conveyed by $s_{t}$ that cannot be obtained from the past information. Note that ${𝒮}_{t}$ is independent of ${ℱ}_{t-1}$, but not of $X_{t}^{*}$, and therefore not of $U_{t}^{*}$.

By contrast to ${ℱ}_{t-1}$, ${𝒮}_{t}$ captures real-time information arriving at time t, after the core prediction was made at t−1, and that cannot be extracted from information set ${ℱ}_{t-1}$. For financial markets, such information can be on order-flow imbalances, intra-day realized volatility, unexpected news releases, and also on relevant economic, political, weather-related, and other news not incorporated into the core prediction. Importantly, the role of information set ${𝒮}_{t}$ is not to shift the mean forecast but to reduce the dispersion of realized forecast errors by possibly triggering stabilizing actions. Empirical studies of forecast errors in financial markets show low correlation between ex-ante forecasts and subsequent real-time information updates, especially during periods of market stress when stabilizing interventions are most common; see, e. g., Fleming and Remolona [20].

It is relevant that the time distance between t and t−1 is short. In daily or intra-day settings, ${𝒮}_{t}$ typically consists of genuinely new microstructure signals or unanticipated shocks that are inaccessible at time t−1, which makes them exogenous to the forecast process. In contrast, when considering lower frequencies, such as monthly, the information environment becomes more dispersed. In such a case, forecast updates are released throughout the month, and policymakers or market participants may directly base their actions on these forecasts. As a result, the chance of feedback between forecasts and stabilization signals increases, making this assumption less credible.

Consequently, and disregarding the expected feedback from the possible policy effect, the prediction augmented by ${𝒮}_{t}$ is $E\left(X_{t}|F_{t-1},{𝒮}_{t}\right)$. Let


$$D_{t}=E(X_{t}|F_{t-1},S_{t})-m_{t}\;and\;Z_{t}=\frac{D_{t}}{\sigma_{t}}$$

be the absolute and relative differences between the augmented and core predictions. Assume that $\left(U_{t}^{*},Z_{t}\right)$ is *iid*, with


$$\left(\;\;\begin{array}{c} {}*20cU_{t}^{*}\\ Z_{t} \end{array}\;\;\right)\sim N\left\{0,\left(\;\;\;\begin{array}{cc} {}*20c1 & \varrho \sigma_{Z}\\ \varrho \sigma_{Z} & \sigma_{Z}^{2} \end{array}\;\;\;\right)\right\}.$$ (1)

When $\left|D_{t}\right|$ is large enough, e.g., it exceeds one standard deviation, the policy makers decide to act in such a way that the actual economic or financial variable becomes


$$X_{t}=X_{t}^{*}+\alpha D_{t}1_{Z_{t}>\nu }+\beta D_{t}1_{Z_{t}<\kappa },$$

where κ≤0≤ν; $1_{Z_{t}>\nu }$ is an indicator of event $\left\{Z_{t}>\nu \right\}$, that is $1_{Z_{t}>\nu }=1$ if $Z_{t}>\nu$ and 0 otherwise; similarly, $1_{Z_{t}<\kappa }$ is an indicator of event $\left\{Z_{t}<\kappa \right\}$. If the policymaker perceives $D_{t}$ as an unexpected shock on the economic or financial variable that needs to be attenuated, the values of α and β are negative. Note that, by scaling the parameters α, β, ν and κ, it is not restrictive to assume $\sigma_{Z}=1$ in (1). The conditional prediction error based on the past is then


$$U_{t}:=\frac{X_{t}-m_{t}}{\sigma_{t}}=U_{t}^{*}+\alpha Z_{t}1_{Z_{t}>\nu }+\beta Z_{t}1_{Z_{t}<\kappa }$$ (2)

The distribution of $U_{t}$ is the *normalized weighted skew-normal* (see Charemza, Díaz and Makarova [15]), where the policy decisions on the distribution of forecast errors are reflected by α and β in (2), representing the policy’s strength. The parameters α and β show the effects of, respectively, the downward and upward correcting action undertaken when the real-time forecast signal $Z_{t}$ exceeds the upper threshold ν, or is below the lower threshold κ.

As in the empirical part of the paper, we are adapting the weighted skew-normal distribution to sovereign bond yields, the parameters α and β have a slightly different interpretation than that in Charemza, Díaz and Makarova [15] model, originally designed for analyzing inflation forecast uncertainty. In our case, the parameter α captures the adjustment when the observed volatility of bond yields is higher than warranted by economic fundamentals, implying that policy acts to reduce excessive uncertainty. In contrast, β would correspond to a correction when observed volatility is lower than underlying risks might suggest. However, in the context of bond market stabilization, the objective of policy is typically not to increase uncertainty, even if it appears understated. Therefore, it is reasonable to set β equal to zero or close to zero, reflecting that interventions are designed to reduce excessive uncertainty rather than actively introduce additional uncertainty. Under this specification, α alone represents the adjustment of forecast uncertainty, and a negative α would indicate a rational stabilization action. Consequently, testing for α=β=0 is of interest, as rejecting this hypothesis confirms the existence of policy footprints in the distribution of forecast errors.

The parameter ϱ, the correlation coefficient between $U_{t}^{*}$ and $Z_{t}$ captures the proportion of the variation in forecast uncertainty that can be statistically attributed to the external information signal. A higher ϱ indicates that real-time information influences the distribution of forecast errors stronger and is more relevant for stabilization policy, compared to the case when ϱ is close to zero.

**Fig 1:**
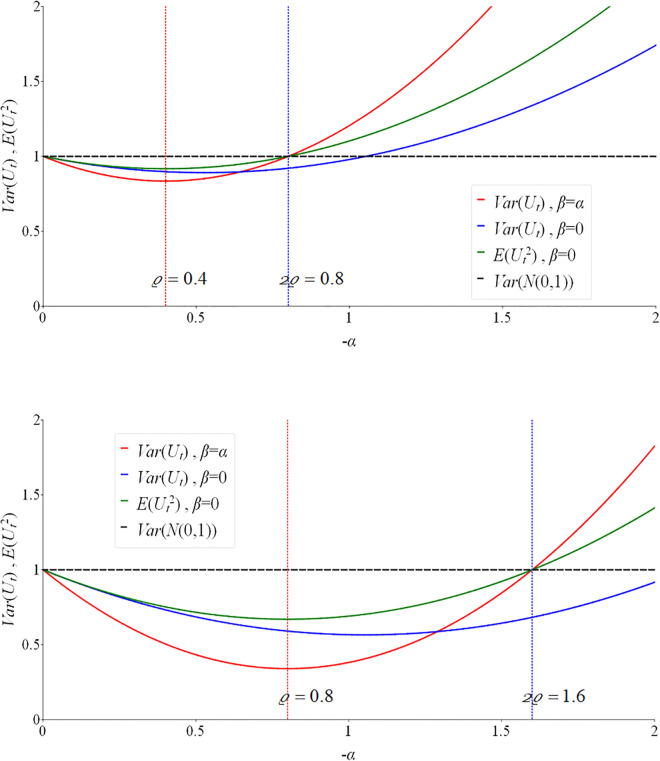
Stabilization policy effects in variance of forecast errors. Legend: the upper panel shows the results for ϱ=0.4 and the lower panel for ϱ=0.8.

Fig 1 demonstrates how economic policies might affect the distribution of ex-post forecast errors. It plots variance $Var(U_{t})$ and $E(U_{t}^{2})$, the second ordinary (non-centered) moment of $U_{t}$, against −α (for better visibility we use −α rather than α). Unlike variance, $E(U_{t}^{2})$ also captures the first-moment effects of the policy. The upper panel of Fig 1 shows the result for ϱ=0.4, that is, for the case where the influence of real-time information on the distribution of forecast errors is reasonably weak, and the lower panel for ϱ=0.8, where such influence is much stronger. Other parameters of the distributions are ν=−κ=1, and either β=0 or β=α for 0<−α<2. If the strengths of the policies are identical regardless of their direction (α=β<0) then $E(U_{t})=0$ and $Var(U_{t})=E(U_{t}^{2})$. If there are no policy effects, then α=β=0 and distribution of $U_{t}$ collapses to the standard normal, which is marked by the dashed horizontal line drawn at 1. The plot shows that the policies, if effective, reduce forecast uncertainty compared to that of the ‘no action’ case (α=β=0) by lowering its variance below 1, if −α<2ϱ. The maximum stabilization effect is achieved at −α=ϱ. However, if the policies are too strong, that is, if −α>2ϱ they become counter-effective, as the variance exceeds that of the ‘no action’ policy. While comparing the upper and lower panels of Fig 1, it can be noticed that with the increase in ϱ, that is, when the influence of real-time information on the distribution of forecast uncertainty becomes stronger, the uncertainty reduction effect increases and is evident for a wider range of α. The blue and green lines in Fig 1 illustrate the extreme asymmetric case where α<0,β=0, in which the first moment of the series is also affected, as $Var(U_{t})\neq E\left(U_{t}^{2}\right)$ Thus, Fig 1 explains the relevance of testing for α=β=0.

In the next section, we consider a general time series model with the WSN innovations and propose the PELM test for the absence of policy interventions; that is, for α=β=0.

## 3 The model and testing

### 3.1 Introducing the PELM test

For the purpose of testing, we consider the stationary process $\left(X_{t}\right)$ satisfying:


$$X_{t}=m(X_{t-1},\ldots ,X_{t-p};\theta_{0m})+\sigma (X_{t-1},\ldots ,X_{t-q};\theta_{0\sigma })U_{t},$$ (3)

where m(·) and σ(·) are functions valued in ℝ and ${\mathbb{R}}^{+}$ respectively, and where $\theta_{0m}$ and $\theta_{0\sigma }$ are vectors of unknown parameters belonging to $\Theta_{m}\subset {\mathbb{R}}^{s_{m}}$ and $\Theta_{\sigma }\subset {\mathbb{R}}^{s_{\sigma }}$. We do not include any exogenous variables in m(·) and σ(·). However, the model with such variables is discussed in Supplement 1 Sections S1 and S5. It is assumed that $U_{t}$ is independent of $\left\{X_{u},u<t\right\}$ and that $\left(U_{t}\right)$ is *iid* WSN distributed with the parameters:


$$\theta_{0u}=(\alpha_{0},\beta_{0},\nu_{0},\kappa_{0},\varrho_{0}{)}^{\prime }\in \Theta_{u}\subset (-\infty ,0{]}^{2}\times [0,\infty )\times (-\infty ,0]\times (-1,1).$$ (4)

Note that, because the WSN is not centered, the functions $m(\cdot ;\theta_{0m})$ and $\sigma (\cdot ;\theta_{0\sigma })$ do not correspond to the first conditional moments of $X_{t}$ (except when $\alpha_{0}=\beta_{0}=0$). This is why parameters $\theta_{0m}$ and $\theta_{0\sigma }$ cannot be estimated by the quasi-maximum likelihood, but must be estimated by the maximum likelihood (ML) together with $\theta_{0u}$. The proof of asymptotic consistency, generalization that allows for adding exogenous variables in (3), and other properties of the ML estimator of $\vartheta_{0}=(\theta_{0m}^{\prime },\theta_{0\sigma }^{\prime },\theta_{0u}^{\prime }{)}^{\prime }$, are provided in Supplement 1, Section S1. Note that our testing approach, as described further, allows us to circumvent the problem of the ML estimation, which is numerically awkward. Nevertheless, the properties of the ML estimator are needed for the derivation of the asymptotic properties of the test.

As discussed in Section 2, we are interested in testing the hypothesis:

$H_{0}:\alpha_{0}=\beta_{0}=0$   against   $H_{1}:\alpha_{0}<0\;\;or\;\;\beta_{0}<0,$ (5)

where $\alpha_{0},\beta_{0}$ are the parameters of the WSN distribution defined in (2) and (4).

Note that, under $H_{0}$, the parameter $\pi_{0}=(\nu_{0},\kappa_{0},\varrho_{0}{)}^{\prime }$ of the WSN distribution is not identified as the distribution of $U_{t}$ collapses to the standard normal; see definition of $U_{t}$ in (2), and Lemma 1 on the properties of the ML estimator of $\vartheta_{0}=(\theta_{0m}^{\prime },\theta_{0\sigma }^{\prime },\theta_{0u}^{\prime }{)}^{\prime }$ does not apply (see Supplement 1 Section S1). Denote by Π the parameter space for $\pi_{0}$. Let $\theta_{0}=(\theta_{0m}^{\prime },\theta_{0\sigma }^{\prime }{)}^{\prime }\in \Theta_{m}\times \Theta_{\sigma }$ be the vector of the parameters that remain unknown under the null. Under $H_{0}$, the WSN density $f_{\theta_{0u}}$ reduces to the density of the standard normal distribution. The maximum likelihood estimator of θ is then defined by


$$\hat{\theta }=\mathrm{\backslash arg}\underset{\theta \in \Theta_{m}\times \Theta_{\sigma }}{\max}{\tilde{Q}}_{n}^{*}(\theta ),\;{\tilde{Q}}_{n}^{*}(\theta )=\frac{\text{1}}{n}\sum_{t=1}^{n}{\tilde{\ell }}_{t}^{*}(\theta ),$$ (6)

where


$${\tilde{\ell }}_{t}^{*}(\theta )=\log\left\{\frac{\text{1}}{{\tilde{\sigma }}_{t}(\theta_{\sigma })}\phi \left(\frac{X_{t}-{\tilde{m}}_{t}(\theta_{m})}{{\tilde{\sigma }}_{t}(\theta_{\sigma })}\right)\right\}=-\frac{\text{1}}{\text{2}}\left\{{\left(\frac{X_{t}-{\tilde{m}}_{t}(\theta_{m})}{{\tilde{\sigma }}_{t}(\theta_{\sigma })}\right)}^{2}+\log{\tilde{\sigma }}_{t}^{2}(\theta_{\sigma })\right\}.$$ (7)

Thus $\hat{\theta }=({\hat{\theta^{\prime }}}_{m},{\hat{\theta^{\prime }}}_{\sigma }{)}^{\prime }$ is the Gaussian MLE of $\theta_{0}$ under $H_{0}$. The strong consistency and asymptotic normality of $\hat{\theta }$ defined by (6) – (7) are given by **Proposition 1** in Supplement 1 Section S2. To distinguish formulas that appear in the Supplement 1 and are referred to in the main text, we use the prefix ’S’ (S1, S2, etc.).

In view of Proposition 1, and in particular (S5), which shows that the Fisher information matrix $I_{\pi }$ is invertible when $\varrho_{0}\neq 0$ and $\left(\nu_{0},\kappa_{0})\neq (0,0\right)$, we now restrict the possible values π to a compact set not including zeros, of the form


$$\Pi_{0}=[\underset{―}{\nu },\overset{―}{\nu }]\times [\underset{―}{\kappa },\overset{―}{\kappa }]\times \left\{\left[-\overset{―}{\varrho },-\underset{―}{\varrho }]\cup [\underset{―}{\varrho },\overset{―}{\varrho }\right]\right\},$$

where $0<\underset{―}{\nu }<\overset{―}{\nu }$, $\underset{―}{\kappa }<\overset{―}{\kappa }<0$ and $0<\underset{―}{\varrho }<\overset{―}{\varrho }$. Now, consider the PELM test statistic


$$PELM_{n,\pi }=n\frac{\partial }{\partial \underset{―}{\theta }u\prime }{\tilde{Q}}_{n}\left\{\hat{\vartheta }(\pi )\right\}{\hat{I}}^{{\underset{―}{\theta }}_{u}}\frac{\partial }{\partial {\underset{―}{\theta }}_{u}}{\tilde{Q}}_{n}\left\{\hat{\vartheta }(\pi )\right\},$$ (8)

where $\hat{\vartheta }(\pi )=({\hat{\theta }}^{\prime },\theta_{u}(\pi {)}^{\prime }{)}^{\prime }=({\hat{\theta }}^{\prime },0,0,\pi^{\prime }{)}^{\prime }$, ${\underset{―}{\theta }}_{u}=(\alpha ,\beta {)}^{\prime }$ and ${\hat{I}}^{{\underset{―}{\theta }}_{u}}$ is the lower-right block of size 2×2 of the matrix ${\hat{I}}_{\pi }^{-1}$, which exists for n large enough when $\pi \in \Pi_{0}$, by (S5). The following Proposition 2 provides details for computing the derivatives in (8), see (S10) – (S11), and summarizes the properties of the $PELM_{n,\pi }$ test statistic.

**Proposition 2** *Under* $H_{0}$ *and the other conditions of Proposition 1, for each* $\pi \in \Pi_{0}$,*we have*


$$PELM_{n,\pi }\overset{d}{\to }\chi_{2}^{2},\;\;as\;\;n\to \infty ,$$

*where* $\chi_{2}^{2}$ *denotes the chi-square distribution with 2 degrees of freedom.*

The proof of Proposition 2 is provided in Supplement 1 Section S3. This Proposition facilitates testing substantially, as the p-values and critical values of the $\chi_{2}^{2}$ distribution can be used, if the statistical sample is large enough (see Section 4 for more detailed results). However, the drawback of using the $PELM_{n,\pi }$ statistic for testing is that it relies on the somewhat arbitrary choice of $\pi \in \Pi_{0}$. To solve a similar problem Davies [21] and Davies [22] suggested using the supremum test statistic


$$PELM_{n}=\underset{\pi \in \Pi_{0}}{\sup}PELM_{n,\pi }.$$ (9)

Supremum Lagrange Multiplier tests are often used for testing nonlinearities in time series; see, e.g., Christou and Fokianos [23]; Francq, Horváth and Zakoïan [24]; Kirch and Kamgaing [25]. Weighted averages of these and other test statistics have also been proposed in the literature (see, e.g., Hansen [26]). Under $H_{0}$ and the other conditions of Proposition 1, $PELM_{n}$ converges to a limit distribution, which justifies approximating the p-values in finite samples by simulation. The full set of properties of $PELM_{n}$ test statistics defined by (9) are formulated and proved in **Proposition 3** in Supplement 1 Section S4.

### 3.2 The PELM test in ARMA-GARCH model

In the empirical part of the paper, in Section 5, we apply the test for one of the most popular dynamic volatility models, the ARMA-GARCH model. In practice, more advanced models with time-varying volatility are often used; see, e.g., Huang, Chiang, and Lin [27], Zou, Xu, and Chen [28], and others, for recent important applications and developments. However, we focus on the ARMA-GARCH model due to its popularity among practitioners and forecasting accuracy, which, in its original form or variations, has frequently proven satisfactory to practitioners (see, for example, Clark and Ravazzolo [29]; Naimy et al. [30]; Wu, Kuang, and Xu et al. [31]).

We also considered other time series models that satisfy (3). In particular, we analyzed the double autoregressive models (see Ling [32], Ling and Li [33]) and, for financial applications, Cai, Montes-Rojas, and Olmo [34]. Although we conducted extensive power and size distortion analysis for these models, we have decided not to use them here, as their interpretation in the context of the sovereign bond market analysis is unclear.

For simplicity, we focus on the first-order ARMA-GARCH model, that is ARMA(1,1)-GARCH(1,1). However, the straightforward generalization holds for higher-order lags in the AR part of the model, which will be utilized in the empirical example in Section 5. Here we discuss the ARMA(1,1)-GARCH(1,1) model given in the form:


$$\begin{array}{c} \left\{ \begin{array}{c} {}*35@lX_{t}=a_{0}X_{t-1}+c_{0}+\epsilon_{t}-b_{0}\epsilon_{t-1}\\ \epsilon_{t}=\sigma_{t}^{*}(\theta_{0\sigma })U_{t},\;\sigma_{t}^{*2}(\theta_{0\sigma })=\omega_{0}+\gamma_{0}\epsilon_{t-1}^{2}+\delta_{0}\sigma_{t-1}^{*2}(\theta_{0\sigma }), \end{array}\right. \end{array}$$ (10)

where $\theta_{0m}=(a_{0},b_{0},c_{0}{)}^{\prime }$ and $\theta_{0\sigma }=(\omega_{0},\gamma_{0},\delta_{0}{)}^{\prime }$. First, note that (10) is not exactly of the form (3) because


$$\sigma_{t}^{*2}(\theta_{0\sigma })=\omega_{0}+\gamma_{0}{\left\{\sum_{i=0}^{\infty }b_{0}^{i}(X_{t-i-1}-a_{0}X_{t-i-2}-c_{0})\right\}}^{2}+\delta_{0}\sigma_{t-1}^{*2}(\theta_{0\sigma }),$$

with $\theta_{0}=(\theta_{0m}^{\prime },\theta_{0\sigma }^{\prime }{)}^{\prime },$ does not depend only on $\theta_{\sigma }$, but also on the mean parameter $\theta_{m}$ via the approximation of the linear innovation ${\tilde{\epsilon }}_{t}(\theta_{m})$, which is recursively defined by


$${\tilde{\epsilon }}_{t}(\theta_{m})=X_{t}-aX_{t-1}-c+b{\tilde{\epsilon }}_{t-1}(\theta_{m}),\;t=2,\ldots$$

for some fixed initial value ${\tilde{\epsilon }}_{1}(\theta_{m})={\tilde{\epsilon }}_{1}.$ However, the results of Propositions 1–3 remain valid, provided that $\theta_{\sigma }$ and ${\hat{\theta }}_{\sigma }$ are replaced by corresponding recursive estimators, which account for this dependency, as shown in (S18) – (S20).

Practical implementation of the two PELM tests in the ARMA-GARCH model can be done in three steps described below (additional explanations and formulas are given in Supplement 1):

1. Estimate the ARMA-GARCH model under the null of (5) by the quasi-maximum likelihood estimate (QMLE) as in (6) – (7).
2. For the $PELM_{n,\pi }$ test, define the parameters $\pi =(\nu ,\kappa ,\varrho {)}^{\prime }$. If the residuals are standardized, the natural settings for ν and κ are + /- one standard deviation of the distribution of the ex-post forecast errors. It would correspond to the intuitive presumption that the forecast errors exceeding one standard deviation can be regarded as ’large’, and hence a stabilization policy should be applied. The setting for ϱ might depend on some external knowledge; the higher ϱ, the greater is the relevance of the real-time information for explaining forecast uncertainty.
3. For the $PELM_{n}$ test, set the upper and lower limits for ν, κ and ϱ that is, intervals $[\underset{―}{\nu },\overset{―}{\nu }],$ $\left[\underset{―}{\kappa },\overset{―}{\kappa }\right]$ and $\left[\underset{―}{\varrho },\overset{―}{\varrho }\right]$. The wider intervals cover more combinations of the parameters and hence are safer to apply; on the other hand, narrower intervals increase the power of the test if they are properly set. Also, decide on the number of drawings from these intervals, as the large number of drawings might slow down computations substantially, while a too small number of drawings would distort the size of the test (see Section 4 further on for a discussion of numerical results which help with this decision).
4. Denote the cumulative function and probability density function of the standard normal distribution by Φ(·) and ϕ(·) respectively. Let also

${\tilde{m}}_{t}(\theta_{m})=m(X_{t-1},\ldots ,X_{1},x_{0},x_{-1},\ldots ;\theta_{m})$ and ${\tilde{\sigma }}_{t}(\theta_{\sigma })=\sigma (X_{t-1},\ldots ,X_{1},x_{0},x_{-1},\ldots ;\theta_{\sigma })$

are empirical approximations of m(·;θ) and σ(·;θ) in the model specification (3). The derivatives for calculating $PELM_{n,\pi }$ appearing in (8) can be computed (as shown by (S10) – (S11)) as

$\frac{\partial {\tilde{Q}}_{n}\left\{\hat{\vartheta }(\pi )\right\}}{\partial {\underset{―}{\theta }}_{u}}=\frac{\text{1}}{n}\sum_{t=1}^{n}g_{\pi }\left({\tilde{U}}_{t}\right)$, where

${\tilde{U}}_{t}=\frac{X_{t}-{\tilde{m}}_{t}({\hat{\theta }}_{m})}{{\tilde{\sigma }}_{t}({\hat{\theta }}_{\sigma })},\;g_{\pi }(x)=(g_{\alpha ,\pi }(x),g_{\beta ,\pi }(x){)}^{\prime },$

and


$$\begin{array}{c} {}*20cg_{\alpha ,\pi }(x)=(x^{2}-1)\varrho \Phi \left(\frac{\varrho x-\nu }{\sqrt{1-\varrho^{2}}}\right)+\phi \left(\frac{\varrho x-\nu }{\sqrt{1-\varrho^{2}}}\right)\left\{\frac{(1-\varrho^{2})x-\nu \varrho }{\sqrt{1-\varrho^{2}}}\right\}\\ g_{\beta ,\pi }(x)=(x^{2}-1)\varrho \Phi \left(\frac{-\varrho x+\kappa }{\sqrt{1-\varrho^{2}}}\right)+\phi \left(\frac{\varrho x-\kappa }{\sqrt{1-\varrho^{2}}}\right)\left\{\frac{\kappa \varrho -(1-\varrho^{2})x}{\sqrt{1-\varrho^{2}}}\right\} \end{array},$$ (11)

Matrix ${\hat{I}}^{{\underset{―}{\theta }}_{u}}$ in (8) is the lower-right block of size 2×2 of the inverse estimated Fisher information matrix, ${\hat{I_{\pi }}}^{-1}$, with the following components (see (S3), (S6) and (S18) – (S20)):


$${\hat{I}}_{\pi }=\left(\begin{array}{cc} {}*20c{\hat{I}}_{\theta } & {\hat{I}}_{\theta ,{\underset{―}{\theta }}_{u}}\\ {\hat{I}}_{{\underset{―}{\theta }}_{u},\theta } & I_{{\underset{―}{\theta }}_{u}} \end{array}\right),\;$$

where $I_{{\underset{―}{\theta }}_{u}}=Eg_{\pi }(U_{t})g_{\pi }^{\prime }(U_{t})$, with $g_{\pi }(\cdot )$ defined by (11); and


$${\hat{I}}_{\theta }=\frac{1}{n}\sum_{t=1}^{n}\frac{1}{{\tilde{\sigma }}_{t}^{2}(\hat{\theta })}\frac{\partial \;{\tilde{\epsilon }}_{t}({\hat{\theta }}_{m})}{\partial \theta }\frac{\partial \;{\tilde{\epsilon }}_{t}({\hat{\theta }}_{m})}{\partial \theta^{\prime }}+\frac{1}{2}\frac{1}{n}\sum_{t=1}^{n}\frac{1}{{\tilde{\sigma }}_{t}^{4}(\hat{\theta })}\frac{\partial {\tilde{\sigma }}_{t}^{2}(\hat{\theta })}{\partial \theta }\frac{\partial {\tilde{\sigma }}_{t}^{2}(\hat{\theta })}{\partial \theta^{\prime }}$$ (12)

and


$$\begin{array}{c} {\hat{I}}_{\theta ,{\underset{―}{\theta }}_{u}}=\frac{-1}{n}\sum_{t=1}^{n}\frac{1}{{\tilde{\sigma }}_{t}(\hat{\theta })}\frac{\partial \;{\tilde{\epsilon }}_{t}({\hat{\theta }}_{m})}{\partial \theta }EU_{1}{g^{\prime }}_{\pi }(U_{1})+\frac{1}{2n}\sum_{t=1}^{n}\frac{1}{{\tilde{\sigma }}_{t}^{2}(\hat{\theta })}\frac{\partial {\tilde{\sigma }}_{t}^{2}(\hat{\theta })}{\partial \theta }E(U_{1}^{2}-1){g^{\prime }}_{\pi }(U_{1}), \end{array}$$ (13)

where the linear innovations ${\tilde{\epsilon }}_{t}(\theta_{m})$ being recursively defined by:

${\tilde{\epsilon }}_{t}(\theta_{m})=X_{t}-aX_{t-1}-c+b{\tilde{\epsilon }}_{t-1}(\theta_{m}),\;t=2,\ldots$.

In (12)-(13) the derivatives are computed recursively (see (S20)) as


$$\frac{\partial \;{\tilde{\epsilon }}_{t}(\theta_{m})}{\partial \theta }=\left(\begin{array}{c} -X_{t-1}\\ {\tilde{\epsilon }}_{t-1}(\theta_{m})\\ -1\\ 0\\ 0\\ 0 \end{array}\right)+b\frac{\partial \;{\tilde{\epsilon }}_{t-1}(\theta_{m})}{\partial \theta },\;\frac{\partial {\tilde{\sigma }}_{t}^{2}(\theta )}{\partial \theta }=\left(\begin{array}{c} 2\gamma \epsilon_{t-1}(\theta_{m})\frac{\partial {\tilde{\epsilon }}_{t-1}(\theta_{m})}{\partial \theta_{m}}\\ 1\\ \epsilon_{t-1}^{2}(\theta_{m})\\ {\tilde{\sigma }}_{t-1}^{2}(\theta ) \end{array}\right)+\delta \frac{\partial {\tilde{\sigma }}_{t-1}^{2}(\theta )}{\partial \theta }.$$

For $PELM_{n}$ test use (9) to obtain a supremum as described in 3 above.

The codes in Aptech Gauss for computing the $PELM_{n,\pi }$ and $PELM_{n}$ are available on GitHub https://github.com/Svetlana2201/Policy-effects.

## 4 Size distortion and power of the tests

This section gives the selected numerical results on the evaluation of size distortion and power of the tests proposed in Section 3. For the analysis of size distortion, we present results obtained for the ARMA-GARCH model (10) with the parameters set as $\left(a_{0},b_{0},c_{0},\omega_{0},\gamma_{0},\delta_{0})=(0.5,0.2,1,1,0.5,0.3\right)$ (results for other ARMA-GARCH settings are available upon request; the outcomes of the size distortion analysis are not substantially affected by changes in ARMA-GARCH specification). These results are obtained by simulation. For the $PELM_{n,\pi }$ test, we set $\alpha_{0}=\beta_{0}=0$ and $\pi_{0}=(\nu_{0},\kappa_{0},\varrho_{0}{)}^{\prime }=(1,-1,0.8{)}^{\prime }$. We apply the $PELM_{n}$ test in two variants, named respectively $PELM_{n}(narrow)$ and $PELM_{n}(wide)$, which differ from each other by the width of the intervals we set for $\nu_{0}$ and $\kappa_{0}$. For the $PELM_{n}(narrow)$ test, we draw $\nu_{0}$ from the interval [0.9,1.1], and $\kappa_{0}$ from the interval [−1.1,−0.9]. For the $PELM_{n}(wide)$ test, the drawings are from intervals of [0.75,1.25] and [−1.25,−0.75] respectively. For all tests, we keep $\varrho_{0}=0.8$.

As in (1)-(2), for $PELM_{n,\pi }$, $\nu_{0}$ and $\kappa_{0}$ are equal to one standard deviation of the distribution of the real-time forecast signals. This choice has been motivated by the common presumption that shocks of less than one standard deviation can be regarded as ’small’, and, hence, likely to be neglected by policymakers. We choose a reasonably high value of the parameter $\varrho_{0}$, as it corresponds to assuming that the real-time information is highly relevant for explaining forecast uncertainty (see Section 2). The supremum in (9) is computed using 40 independent draws from these intervals. The number of replications is equal to 10,000 for each run. For the $PELM_{n}$ test, the practical problem is to decide on the number of drawings made from the intervals $\left[\underset{―}{\nu },\overset{―}{\nu }\right]$, $\left[\underset{―}{\kappa },\overset{―}{\kappa }\right]$ and $\left[\underset{―}{\varrho },\overset{―}{\varrho }\right]$, as, when the number of drawings increases, the computation time increases substantially. Consequently, we conducted some additional simulation experiments and found that increasing this number above 40 does not alter the estimated size of the test in any systematic way. This conclusion stems from the results of the randomness test of the quantiles of the $PELM_{n,\pi }$ statistic obtained for the case where the number of drawings is gradually increased (see Supplement 2 Part 1). They show that, when the number of drawings exceeds 40, the null hypothesis that the estimated size of the test changes randomly rather than systematically cannot be rejected. Also, the rate of the standard deviations to the means of the computed quantiles of the $PELM_{n}$ test statistics obtained for a different number of drawings are in the range of 0.5–3.0%, which is quite small. Hence, increasing the number of drawings above 40 does not add any meaningful information on the true test size. Therefore, to speed up computations, we have decided to use 40 as the number of such drawings.

**Fig 2:**
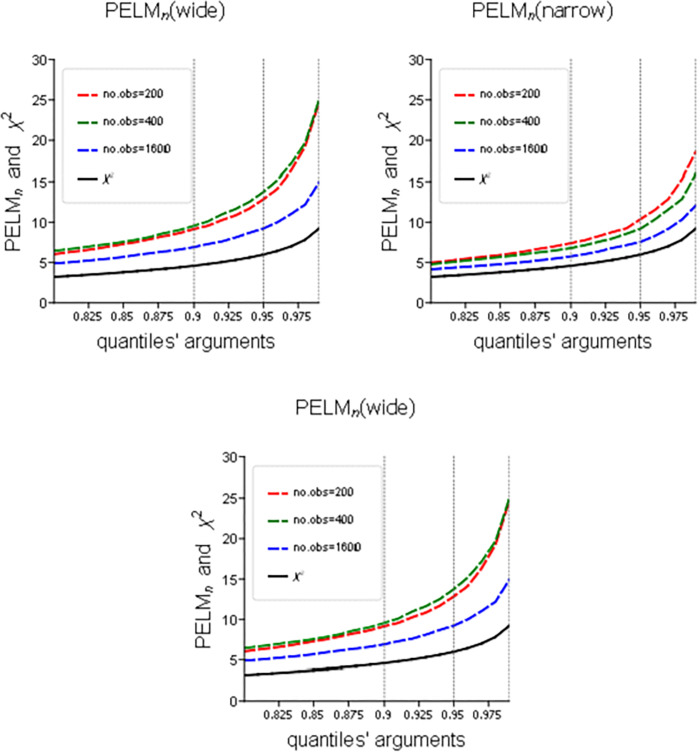
Size distortions in $PELM_{n,\pi }$, $PELM_{n}(narrow)$, and $PELM_{n}(wide)$ test statistics (right tails of the simulated inverted CDFs).

Fig 2 shows the right tails of the simulated inverted CDFs of the distributions of the $PELM_{n,\pi }$ and $PELM_{n}$ statistics that are computed under the null for different sample sizes, with 10,000 simulations in each case. It also shows the right tail of the $\chi_{2}^{2}$ CDF with the quantiles $q_{0.8}$, $q_{0.9}$ and $q_{0.95}$ marked by vertical dotted lines. It demonstrates the downward bias if the $\chi_{2}^{2}$ p-values or critical values are used instead of the true but unknown ones. Such bias is not substantial for the $PELM_{n,\pi }$ test, particularly for the quantiles smaller than 0.95, which is the conventional level of significance. However, it is slightly bigger for $PELM_{n}(narrow)$ and much bigger for $PELM_{n}(wide)$.

For a quick evaluation of the significance, we constructed Table 1, with the most frequently used critical values that correspond to selected quantiles. For comparison, the corresponding $\chi_{2}^{2}$ critical quantiles are also included. This table is essentially an excerpt from Fig 2, but its traditional critical values form makes it straightforward to use.

**Table 1:** Simulated quantiles for $PELM_{n,\pi }$, $PELM_{n}(narrow)$ and $PELM_{n}(wide)$ test statistics.

Probability	$\chi_{2}^{2}$ quantiles	Sample size		
		200	400	1600
		$PELM_{n,\pi }$ quantiles		
0.800	3.219	4.280	4.137	3.805
0.850	3.794	5.073	4.944	4.391
0.900	4.605	6.308	5.942	5.247
0.950	5.991	8.550	7.971	6.993
0.990	9.210	15.570	13.150	10.980
		$PELM_{n}(narrow)$ quantiles		
0.800	3.219	4.980	4.776	4.156
0.850	3.794	5.935	5.684	4.782
0.900	4.605	7.401	6.790	5.756
0.950	5.991	10.360	9.182	7.551
0.990	9.210	18.590	15.960	12.080
		$PELM_{n}(wide)$ quantiles		
0.800	3.219	6.029	6.436	4.902
0.850	3.794	7.284	7.561	5.689
0.900	4.605	9.146	9.552	6.921
0.950	5.991	12.830	13.740	9.225
0.990	9.210	24.710	25.010	14.920

For a more accurate approximation of the p-values, we run Monte Carlo simulations for different sample sizes and settings for π, and then interpolate. We based our approximations on the estimation of 35 different data-generating processes with different sample sizes n (n=200,400,600,1000,1600,3500), and intervals for π set as for $PELM_{n}(narrow)$ and $PELM_{n}(wide)$ above. For the given value of the PELM statistic, we interpolated between adjacent sample sizes and accounted for some (very infrequent and small in magnitude) non-monotonicity by smoothing using the Bézier spline. As a result, we obtained impulse surfaces that produce the p-values with reasonable accuracy.

**Fig 3:**
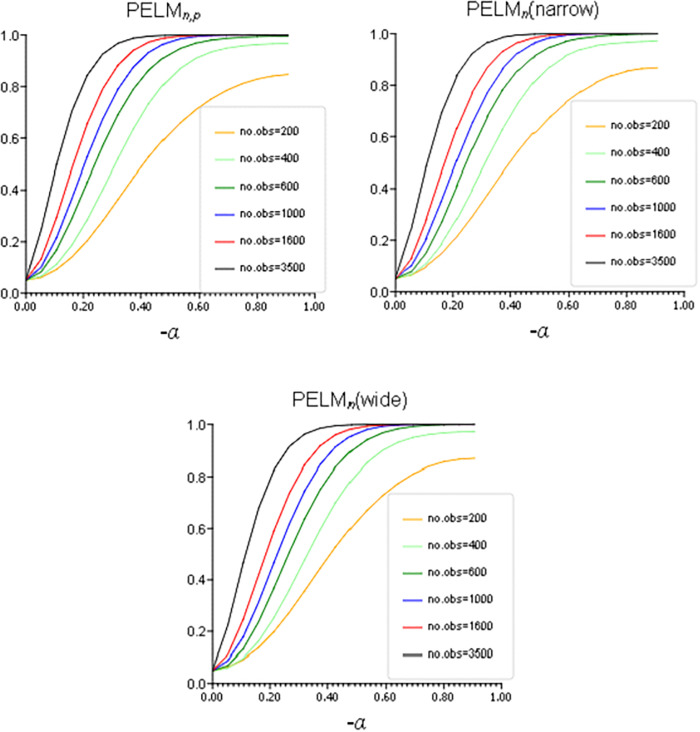
Power of $PELM_{n,\pi }$, $PELM_{n}(narrow)$ and $PELM_{n}(wide)$ for different sample sizes.

We illustrate the evolution of power of the tests for different sample sizes. The settings for simulations are the same as for the analysis of size distortion, except for removing the null hypothesis setting of $\alpha_{0}=\beta_{0}=0$. Fig 3 shows the probabilities of rejecting the null hypothesis for all three tests, that is $PELM_{n,\pi }$, $PELM_{n}(narrow)$ and $PELM_{n}(wide)$, under the alternative hypothesis, where $\alpha_{0}$ is gradually decreasing from zero to −1 and $\beta_{0}=0$. It demonstrates that the power of the tests is monotonously increasing with the increase in the strength of the economic policy shown by $-\alpha_{0}$.

Results of the more detailed simulation analysis (not presented here and available on request) show the power of the $PELM_{n,\pi }$ and $PELM_{n}$ tests do not increase monotonously with the increase in $-\alpha_{0}$ and $-\beta_{0}$, and so with the increase in the policy strength for all values of $\alpha_{0},\beta_{0}\in (-\infty ,0)$. The intuition of this is demonstrated by Fig 1 in Section 1 above. As the variance of $U_{t}$ initially decreases with the decrease in $\alpha_{0}$ and $\beta_{0}$, and then increases, it is prudent to restrict the range of the admissible values of $\alpha_{0}$ and $\beta_{0}$ such as $-\alpha_{0}\in [0,-\alpha^{*}]$ and $-\beta_{0}\in [0,-\beta^{*}]$, where:


$$(\alpha^{*},\beta^{*})=arg\underset{\alpha_{0},\beta_{0}}{\min}Var(U_{t}).$$

It can be shown that $-\alpha^{*}=-\beta^{*}=\varrho_{0}$ for the case where $\nu_{0}=-\kappa_{0}$. However, if there is a lack of symmetry between $\nu_{0}$ and $\kappa_{0}$, so $\nu_{0}\neq -\kappa_{0}$, the relationship can be more complex.

Further results show that the power of the test is reasonably robust to changes of the threshold parameters $\nu_{0}$ and $\kappa_{0}$. Analogously, if $\varrho_{0}$ rises above 0.8, the power is increased accordingly.

## 5 Empirical application: Can the PELM tests help in predicting fiscal improvement?

If our approach is valid, policy imprints on the bond market should be detectable by the PELM test when applied to time series of government bond yields. If these policies were effective, this should also be reflected in positive movements in relevant economic aggregates, such as reductions in budget deficits.

In this context, we formulate the following working hypothesis:

*If a global shock affects the financial stability of some countries, the negative consequences of such a shock are more likely to diminish in countries where imprints of economic policies have been detected in the bond market than in countries where such imprints are absent.*

To test this hypothesis, we compute the $PELM_{n,\pi }$ and $PELM_{n}$ statistics for time series of data on government bond yields for a broad group of countries. We then divide these countries into two groups: Group A, where the PELM test statistics are statistically significant at a reasonable level of significance (i.e., countries where imprints of policies are confirmed by rejecting the null hypothesis in (5)), and Group B, where the test statistics are not significant. To enable cross-country comparison, we focus on possible consequences of a global shock that might affect countries at approximately the same time, albeit in different ways. As the effects of policy might occur after some time, we define the *aftershock year* as the year in which the results of such a shock are likely to become evident for all selected countries, and the *final year* as the year in which the positive effects of the economic counteractive policy are expected to be evident. On factors affecting the timing of the effects of such policy, see Blanchard and Perotti [35]; Mishkin [36]; Reinhardt and Rogoff [37].

We then compare the IMF data on budget deficits as a percentage of GDP for the aftershock and final years. Changes in the budget deficit serve as an indicator of fiscal efficiency. If our hypothesis holds, for countries in Group A where traces of economic policy are found, the differences in budget deficits between the aftershock and final years should be significantly positive; that is, the budget deficit should, on average, decrease. For comparison, we also apply other fiscal indicators, obtaining similar results (see Supplement 2, Part 4).

Our primary example focuses on the effects of policy reactions to the Russian financial crisis that began in mid-2014, culminating on December 16, 2014, and resulting in a drastic devaluation of the Russian ruble. We chose this crisis as a subject of testing rather than the more severe European sovereign debt crisis of 2010−2012 for pragmatic reasons. As we compare budget dynamics across 33 countries, the Russian crisis affected a greater number of countries than the European sovereign debt crisis, making statistical testing more robust. We have not considered the COVID-19 crisis, as its causes were non-financial, its implications were much broader, and it does not fit most definitions of a financial crisis (see, e.g., Babecký et al. [38], for a review of such definitions, and Hu et al. [39], for the analysis of the COVID-19 crisis).

The Russian financial crisis of 2014 developed in mid-2014 and culminated on December 16, 2014, resulting in approximately 50% devaluation of the ruble against the US dollar. This crisis stemmed from two primary factors: international sanctions following Russia’s annexation of Crimea and a sharp decline in oil prices from over $100 to below $60 per barrel. The Central Bank of Russia’s intervention, raising interest rates to 17%, proved insufficient to stabilize the currency, necessitating direct government financial support measures. For a detailed description of the causes and immediate effects of the crisis in Russia, see Dreger et al. [40]; see also Ahmad, Mishra, and Daly [41].

Global sovereign bond markets in 2015–2016 showed varying responses to this crisis. Developed economies experienced yield compression as investors sought low-risk assets (See Bank of International Settlements Annual Report [42]). Emerging markets reacted differently, depending on their economic fundamentals and commodity exposure. Nations heavily dependent on hydrocarbon exports saw significant yield increases, while economies with diversified export structures maintained relatively stable borrowing costs. Countries that had implemented precautionary stabilization policies by maintaining adequate foreign exchange reserves, establishing fiscal buffers, adopting flexible exchange rate mechanisms, and pursuing economic diversification showed greater resilience in their bond markets during this period. These preventive measures created identifiable policy signals in bond markets prior to the crisis (see Demirer, Ferrer, and Shahzad [43]; Costantini and Sousa [44]).

The Russian ruble crisis can only be partly seen as a reflection of a global financial crisis. On the one hand, it shows several characteristics typical of systemic events: a rapid currency decline, sudden halts in capital inflows, increased volatility in the bond market, and fiscal stress affecting many other countries. Its spillover effects spread worldwide through trade, remittances, and financial connections. From this perspective, the crisis illustrates how a shock in one country, exacerbated by external factors such as falling oil prices, can spread through sovereign debt markets.

On the other hand, its global reach was relatively limited in comparison to the 2008 global financial crisis or the European sovereign debt crisis. The most direct effects were geographically concentrated, with fiscal consequences strongest in Russia and oil-dependent economies, while distant advanced economies experienced only mild second-round volatility. In addition, the crisis was compounded by sanctions and commodity price shocks, which made it less “pure” as a financial crisis. As such, its representativeness is restricted; however, its timing, clarity of onset, and measurable fiscal impacts make it valuable for methodological purposes. In this sense, the Russian crisis can serve as a reasonably instructive, though maybe not fully representative, test case for evaluating the stabilization effects of discovering policy footprints.

Being aware of these limitations, we additionally tested our working hypothesis, focusing on the effect of other financial crises that had worldwide implications, namely the crisis triggered by the unexpected outcome of the Brexit referendum in the UK in June 2016. These results are briefly discussed in Section 6 and documented in Supplement 2, Part 4.

We define the aftershock year as 2015, the year when the fiscal consequences of the crisis became evident, and the final years as 2017, 2018, and 2019, when the results of the eventual fiscal policy actions should affect the budget deficit. Using these years allows us to capture the full policy adjustment cycle and avoid blurring the policy effects by the COVID-19 pandemic.

For testing, we use daily data on the yields on government bonds taken from the Thomson Reuters database (http://thomsonreuters.com/). We collected data for 33 countries: Austria, Australia, Belgium, Brazil, Canada, Czech Republic, Chile, Denmark, Finland, France, Germany, Hungary, Iceland, India, Indonesia, Ireland, Italy, Japan, Korea, Mexico, Netherlands, Poland, Portugal, Russia, South Africa, Spain, Slovakia, Slovenia, Sweden, Switzerland, Turkey, United Kingdom, United States. We estimate the ARMA-GARCH models using data from the three-year period 2012–2014, using 783 observations for each country. Estimating the model for this period avoids contaminating the policy efforts by overlapping with the aftermath of the 2008–2009 crisis while capturing relevant pre-2014 crisis stability actions. To avoid, or at least reduce, sample selection bias and enable testing the budgetary effects, we use the broadest possible selection of countries for which data on session-to-session government bond yields are available in the Thomson Reuters database (http://thomsonreuters.com/) and that meet the following criteria.

1. Are complete for the estimation period (1 January 2012−31 December 2014).
2. Data for the IMF indicators for 2017–2019 are also available.
3. Data shows sufficient variability; that is, it does not contain duplications for a reasonably long period of time, or it does not have missing observations.

All relevant data is included in Supplement 3; see also data repository *figshare* at https://figshare.com/articles/dataset/Data_for_Testing_Footprints_xlsx/30016453?file=57852376

In Supplement 2 Parts 2 and 3, we show some descriptive statistics for the data and present results of unit root testing, which confirms stationarity of the data used for the ARMA-GARCH estimation.

Such a panel of data is potentially heterogeneous, as some countries might have conducted stabilization policies in the period investigated, while others might not. Hence, we estimate the ARMA(k,1)-GARCH(1,1) model under the null of (5) using the quasi maximum likelihood method given in (6) – (7). The optimal k was obtained by applying the Ljung-Box criterion, where k is equal to the minimum number of lags for which the Ljung-Box test does not reject the null hypothesis of a lack of autocorrelation in the residuals at the 5% level of significance.

As in Section 3, we apply the $PELM_{n,\pi }$ test with $\nu_{0}=-\kappa_{0}=1$ and $\varrho_{0}=0.8$. While setting of $\nu_{0}$ and $\kappa_{0}$ at the levels of one standard deviation seems to be rational, the choice of $\varrho_{0}$ might be more difficult. As stated in Section 2, it represents the strength of the influence of real-time information on the distribution of forecast errors. Such strength, in practice, might not be easy to assess, as it would require access to detailed and often confidential data supported by some textual analysis and a thorough behavioural understanding of the market-related decision-making mechanism. Due to these complexities, we leave it for further research and assume a priori the reasonable strength of the influence of such information, which allows us to maintain a relatively high power of the test applied (see Section 4).

We use two versions of $PELM_{n}$, namely $PELM_{n}$ (narrow), where the limits on ν and κ needed for computing the supremum in (9) are within the ±0.1 intervals around $\nu_{0}=-\kappa_{0}=1$; and $PELM_{n}$ (wide), with limits of ±0.25 intervals around $\nu_{0}=-\kappa_{0}=1$. We are setting ρ=0.8, as for the $PELM_{n,\pi }$.

Next, we divide the countries into two groups. Group A comprises countries where the PELM statistics are significant at the 0.05 nominal level of significance; that is, countries where we found statistical confirmation of the stabilization policy. Other countries with non-significant PELM statistics are left in Group B. The selection for Groups A and B turned out to be identical for the $PELM_{n,\pi }$ and $PELM_{n}(narrow)$, but it was slightly different if the selection was based on $PELM_{n}(wide)$. For all tests, countries in Group B are Austria, Brazil, France, Korea, Mexico, South Africa, Spain, Turkiye, and the United Kingdom. Under the $PELM_{n}(wide)$ classification, Canada is also in Group B. For the remaining countries, the PELM statistics are significant at the 0.05 level, and these countries are in Group A.

While testing simultaneously for the effects of an economic policy across 33 countries, we effectively conducted 33 separate hypothesis tests. Such simultaneous testing increases the probability of Type I errors (rejecting a true null hypothesis) due to the increased likelihood of obtaining significant results by chance alone (the multiple hypothesis testing problem). We alleviate this problem by applying the Truncated Product Method (TPM); see Zaykin et al. [45]. The TPM is a technique designed to combine p-values in multiple hypothesis testing while emphasizing significant results below an a priori given truncation threshold τ. It helps mitigate the false discovery rate (FDR), which is the expected proportion of false positives among all discoveries, by focusing on p-values below the threshold and reducing noise from non-significant results. The selection of τ influences the ability to detect false positives: lower thresholds mean that only the most significant p-values are combined, increasing sensitivity to true effects but potentially inflating the risk of overlooking genuine but weaker signals.

Table 2 presents the expected proportion of false discoveries of significance across all series by all three PELM tests obtained by TPM for the p-values of the PELM statistics for countries selected for Groups A and B. We also applied more general methods, namely the Modified Truncation Product Method and its bootstrapped counterparts; see Sheng and Yang [46]. The outcomes of these tests are not substantially different, in their essence, from those in Table 2.

**Table 2:** Expected proportions of false discoveries of the significance.

Truncation threshold	$PELM_{n,\pi }$		$PELM_{n}(narrow)$		$PELM_{n}(wide)$	
	Group A	Group B	Group A	Group B	Group A	Group B
τ =0.125	0.000	1.000	0.000	0.117	0.000	0.131
τ =0.100	0.000	1.000	0.000	1.000	0.000	0.131
τ =0.075	0.000	1.000	0.000	1.000	0.000	0.131
τ =0.050	0.000	1.000	0.000	1.000	0.000	1.000

Legend: truncation parameter *τ* indicates the minimum p-values excluded from joint testing.

As it follows from Table 2, for Group A, the TPM consistently produces an extremely small expected proportion of false discoveries, practically equal to zero, across all tests and truncation thresholds, providing strong evidence against the null hypothesis and supporting the claim that the policy footprints have been found for these countries. In contrast, Group B yields large p-values (mostly 1.000) across all tests and truncation values, suggesting no strong evidence to reject the null hypothesis of the lack of presence of such footprints. This pattern holds even at the strictest truncation threshold τ =0.050. Consequently, we can conclude that we have confirmation of the rationale behind the applied split of countries into Groups A and B.

The significance of the PELM statistics is only a necessary, but not sufficient, condition for claiming that our test indeed evidences a stabilization policy. The next step is to evaluate the working hypothesis that the economic policies, if effective, should mitigate the effect of the shock and, consequently, cause a reasonably quick fiscal recovery. This can be confirmed by an’after treatment’ statistical test, which, in our case, is the paired difference test. The test checks whether the differences in the budget deficit to GDP ratio (see http://www.imf.org/external/ns/cs.aspx?id=262) between the aftershock and final years for countries in each Group are statistically positive; that is, the budget deficit diminishes. If this is the case for Group A, but not for Group B, we may conclude that the’treatment’, if discovered by the PELM tests, indeed evidences the presence of an effective stabilization policy.

Table 3 shows the results of this test: the t -ratios of the paired difference test statistics between the aftershock and final years for Groups A and B. The bootstrapped p-values are given in brackets below the test statistics. The results in Table 3 confirm our working hypothesis and, hence, the rationale of our testing method. For Group A, the paired t -test statistics are significant at any sensible level of significance; for Group B, the test statistics are either insignificant or on the verge of 10% significance. Consequently, for countries in Group A, it can be concluded that the economic stabilization policies undertaken prior to 2015 (the aftershock year) are evidenced by the significance of the $PELM_{n,\pi }$ and $PELM_{n}$ statistics, resulting in success, that is, in reduction in budget deficits in later years. As this cannot be said for Group B, we may conclude that our working hypothesis is confirmed.

**Table 3:** Results of paired difference testing.

Selection by	Group A	Group B
Final year 2017		
$PELM_{n,\pi }$ & $PELM_{n}(narrow)$	15.201 (0.000)	−1.085 (0.651)
$PELM_{n}(wide)$	15.193 (0.002)	−1.224 (0.664)
Final year 2018		
$PELM_{n,\pi }$ & $PELM_{n}(narrow)$	28.717 (0.000)	0.460 (0.448)
$PELM_{n}(wide)$	27.185 (0.000)	0.984 (0.396)
Final year 2019		
$PELM_{n,\pi }$ & $PELM_{n}(narrow)$	25.714 (0.000)	0.541 (0.431)
$PELM_{n}(wide)$	24.262 (0.000)	1.194 (0.363)

Legend: columns 2 and 3: the *t*-ratios of the paired difference test statistics between the aftershock and final year; the bootstrapped p-values are in parentheses.

Overall, the findings of this section are promising. We can say that if footprints of economic policy are detected by the PELM test in a country’s sovereign bond yield time series, the chances for a quicker recovery from the crisis increase compared to when such footprints are not confirmed. It is worth noting that the time series used ended before the crisis began. Therefore, the assessment of the recovery chances can be made at the very start of the crisis. In other words, the answer to the question posed in the section’s title, whether the PELM tests can help in predicting fiscal improvement, is yes.

So far, these findings are limited to evidence from one financial crisis and singe fiscal stability indicator. The next Section 6 presents a wider evidence.

## 6 Robustness of the empirical results

The settings of models that give the results described above have been subjected to various robustness checks. We verified whether a different classification of countries into Groups A and B, achieved by excluding certain countries from the sample or altering the size of Group A, affects the validity of our working hypothesis. We also examined the effects of applying a different bootstrapping technique to compute the p-values on the outcome of testing the working hypothesis.

Below, we present a summary of the robustness analysis, with detailed results available upon request.

### 6.1. Sample size sensitivity

We examined how sample size affects estimation outcomes. While our main results use three years of daily data on sovereign bond yields, we tested periods of different lengths: an extended four-year period (2011−2014, 1,048 observations) and two shorter periods: one with two years of data (2013−2014, 524 observations) and another covering eighteen months (mid-2013–2014, 394 observations). Although extending the sample produced similar testing results, we focused on the three-year period to avoid contamination from the earlier financial crisis. The shorter samples produced slightly less significant results in paired difference testing for Group B.

### 6.2. Flexibility of groups’ classification

We tested the sensitivity of our results to group classification by systematically moving countries from Group A to Group B, starting with those showing the least significant PELM statistics (highest p-values). The reclassification of up to five countries from Group A to B maintained the significance of the paired t -test, supporting our working hypothesis.

### 6.3. Time-varying parameter analysis

We implemented a rudimentary time-varying parameters approach using rolling windows of 250 observations for the ARMA-GARCH model estimation. We calculated PELM statistics for each window and identified Group A using discounted sums of cases where PELM statistics showed p-values of 0.01 or less. Using daily data from early 2011, we applied various time discounting methods (see Laibson [47]; Heilmann [48]), weighting recent observations more heavily than earlier to achieve the time preference effect. The pairwise testing results continued to support our working hypothesis.

### 6.4. Alternative fiscal balance indicators

Supplement 2 Part 4, outlines the results of applying our two-step testing approach and using, instead of the budget deficit, three alternative indicators of fiscal imbalance published by the IMF. These indicators are the difference between budget revenue and expenditures, net lending/borrowing, and primary net lending/borrowing. For all three of these indicators, the paired *t*-test statistics are significant, or on the verge of the 10% significance level, for Group A. For Group B, two of these statistics are highly insignificant. Although these results are only fragmentary, they generally confirm the robustness of our approach.

### 6.5. Another example: the effect of the Brexit referendum

As an additional example, we use the effects of the unexpected outcome of the U.K. Brexit referendum, which took place on 23 June 2016. The Brexit referendum shock occurred on June 24, 2016, when the “Leave” victory triggered immediate financial market turmoil. The British pound fell to a 31-year low, equity markets plunged globally, and safe-haven flows intensified toward German bonds and gold. European banks were particularly affected due to their UK exposure, while peripheral eurozone countries faced renewed sovereign stress. For a literature review and comprehensive theoretical analysis, see Drinkwater and Jennings [49].

Both the Russian ruble and Brexit referendum crises shared some common elements. The Russian crisis exhibited classic emerging market vulnerability patterns: currency depreciation, reserve depletion, and contagion to similar economies. The Brexit crisis was characterized by typical uncertainty-driven market responses: a rush to sell riskier assets, currency volatility, and an immediate reassessment of cross-border investment risks. Both crises demonstrated how integrated global financial markets transmit localized shocks internationally through investor sentiment and portfolio rebalancing.

A summary of the testing results for the crisis caused by the Brexit referendum outcome is provided in Supplement 2, Part 4. It shows that the paired *t*-test, which uses the budget deficit data, gives insignificant results for Groups A and B, contradicting our working hypothesis. However, testing the changes in the other three financial balance indicators confirms it; the paired *t-*statistics are significant for Group A and insignificant for Group B. The discrepancy may stem from different methods used to construct these indicators. The insignificant results when testing with the budget deficit in Group A after the Brexit crisis likely reflect this measure’s inclusion of interest payments and one-off items, which are unrelated to uncertainty shocks. These payments, during the immediate post-referendum period, added measurement noise that hid potential stabilization effects. The significance of the paired *t*-test for Group A, while using the other three indicators, suggests that these measures better capture the effects in countries with stronger policy footprints in forecast uncertainty.

## 7 Conclusions

The PELM test we propose in this paper for detecting traces of economic policies in forecast errors of time series models demonstrates, in all its versions, sound asymptotic properties and high power across a wide range of alternatives. Its important advantage is the ability to detect traces of stabilization policy without the need to use additional information on the details of such policy, like textual data or dummy variables. While computationally feasible, its less restrictive version requires combining maximum likelihood estimation with simulation. Nevertheless, the table of critical values given in the text and extensive impulse surface approximations available on request make testing feasible in empirical models. The ARMA-GARCH model we discuss here is reasonably simple, and more advanced alternatives are available.

Our empirical application to modelling 10-year government bond yields produces easily interpretable results. The test proves useful for predicting fiscal balance indicators. Significant PELM test statistics computed for bond yields in a given period serve as a good indicator that the country’s fiscal position might improve in the years ahead. The results are reasonably robust, so our approach can have wider applications in evaluating the effects of stabilization policy.

Although our abductive reasoning approach, understood as forming the most likely explanation from incomplete information, fits a broader tradition in economics that seeks to recover unobserved mechanisms from observable outcomes, it is not within methodological limitations. As is often the case in empirical economics, research based on abductive reasoning is never complete. In our case, other plausible explanations may exist for the significance of the PELM testing and changes in fiscal balance indicators. Hence, further inference on discovering policy footprints in forecast uncertainty is needed, possibly by developing further statistical tests and providing wider empirical evidence.

The test offers several promising applications beyond our current scope. Particularly, it can be applied to discover the stabilization effects of inflation-targeting economic policy and further develop the optimal policy perturbation model. Other potential applications include empirical evaluation of direct and indirect outcomes of forecast-targeting economic policy.

The successful verification of our working hypothesis has further practical consequences, particularly in the context of modern Bayesian forecasting of financial balances (see Martin et al. [50]). Before forecasting financial balances, our PELM test can be applied to time series of government bond yields and other relevant financial indicators. If the test confirms the traces of an economic policy in forecast errors, the priors applied for forecasting can be relatively stronger (with higher weights) than in cases where such effects are not confirmed. Our test can also benefit frequentist or non-econometric forecasting. When the test indicates that economic policy stabilizes the bond market, non-econometric predictions or scenarios can be formulated more confidently.

## Supporting information

Supplement 1 Main proofs, formulas and generalization. (PDF)

Supplement 2 Additional simulation and empirical results. (PDF)

Supplement 3 Data used for empirical examples and robustness analysis. (XLSX)
