# Supplementary material for: Testing for the footprints of stabilization economic policy in forecast errors
Source: PLoS One. 2025 Dec 1;20(12):e0336495. doi: 10.1371/journal.pone.0336495 (PMC12668634; doi:10.1371/journal.pone.0336495)
Supplement: Supplement 1 — (PDF) [file pone.0336495.s001.pdf]

Testing for the footprints of stabilization economic policy in forecast errors

Wojciech Charemza[1,2], Christian Francq[3], Radu Lupu[4], Svetlana Makarova*[5], and Jean-Michel Zakoïan[3]

[1]Vistula University, Poland
[2]University of Leicester, UK
[3]CREST, Paris, France
[4]Bucharest University of Economic Studies and Institute for Economic Forecasting, Romanian Academy, Romania
[5]University College London, UK

SUPPLEMENT 1

Main proofs, formulas and generalization

*Corresponding author: SSEES, 16 Taviton Street, WC1H 0BW, London, UK; s.makarova@ucl.ac.uk

SUPPLEMENT 1: Main proofs, formulas and generalization

In this Supplement, we refer to formulas given in the text by their numbers. In some cases, we repeat the formulas from the main text, keeping their original number. To distinguish formulas that appear in the Supplement, we use the prefix 'S' (S1, S2, etc.). ■

S1. Generalization: Introducing exogenous variables to (3) and properties of the maximum likelihood estimator of parameters of generalized model (3)-(4)

Model (3)-(4) can be generalized by introducing an exogenous variables in (3) and keeping the assumptions about U_t as in (4). More precisely, let (X_t) be a stationary process satisfying

$$X_t = m(X_{t-1}, \dots, X_{t-p}; \theta_{0m}) + \sigma(X_{t-1}, \dots, X_{t-q}; \theta_{0\sigma})U_t, \tag{S1}$$

where where $\boldsymbol{X}_t = (X_t, \boldsymbol{Y}_t')'$ when a vector $\boldsymbol{Y}_t$ of relevant economic and financial variables are available at time t, or simply $\boldsymbol{X}_t = X_t$ when there is no exogenous variable; $m(\cdot)$ and $\sigma(\cdot)$ are functions valued in $\mathbb{R}$ and $\mathbb{R}^+$ respectively, and where θ_{0m} and $\theta_{0\sigma}$ are vectors of unknown parameters belonging to $\Theta_m \subset \mathbb{R}^{s_m}$ and $\Theta_\sigma \subset \mathbb{R}^{s_\sigma}$.

Given the observations $X_1, \dots, X_n$ and $\boldsymbol{Y}_1, \dots, \boldsymbol{Y}_n$, the initial values $\boldsymbol{X}_0 = x_0, \boldsymbol{X}_{-1} = x_{-1}, \dots$ and the parameters $\theta_m \in \Theta_m$ and $\theta_\sigma \in \Theta_\sigma$, for $t = 1, \dots, n$, the functions

$$m_t(\theta_m) = m(\boldsymbol{X}_{t-1}, \boldsymbol{X}_{t-2}, \dots; \theta_m) \quad \text{and} \quad \sigma_t(\theta_\sigma) = (\sigma \boldsymbol{X}_{t-1}, \boldsymbol{X}_{t-2}, \dots; \theta_\sigma)$$

can be approximated by

$$\widetilde{m}_t(\theta_m) = m(\boldsymbol{X}_{t-1}, \dots, \boldsymbol{X}_1, x_0, x_{-1}, \dots; \theta_m), \quad \widetilde{\sigma}_t(\theta_\sigma) = \sigma(\boldsymbol{X}_{t-1}, \dots, \boldsymbol{X}_1, x_0, x_{-1}, \dots; \theta_\sigma).$$

Let $\Theta = \Theta_m \times \Theta_\sigma \times \Theta_u$ be the parameter space of the vector $\vartheta_0 = (\theta_{0m}', \theta_{0\sigma}', \theta_{0u}')'$ of all the unknown parameters. The maximum likelihood estimator (MLE) of ϑ_0 is defined by

$$\widehat{\vartheta} = \arg\max_{\vartheta \in \Theta} \widetilde{Q}_n(\vartheta), \quad \widetilde{Q}_n(\vartheta) = \frac{1}{n}\sum_{t=1}^{n} \widetilde{\ell}_t(\vartheta) \tag{S2}$$

where

$$\widetilde{\ell}_t(\vartheta) = \log\left\{\frac{1}{\widetilde{\sigma}_t(\theta_\sigma)} f_{\theta_u}\left(\frac{X_t - \widetilde{m}_t(\theta_m)}{\widetilde{\sigma}_t(\theta_\sigma)}\right)\right\}.$$

Let U be a random variable with density $f = f_{\theta_{0u}}$. The Fisher information given by an observation $\sigma U + m$ on the location-scale parameter (m, σ) is

$$\frac{1}{\sigma^2}\begin{pmatrix} \iota_m & \iota_{m\sigma} \\ \iota_{m\sigma} & \iota_\sigma \end{pmatrix},$$

with

$$\iota_m = \int \frac{\{f'(x)\}^2}{f(x)}dx, \quad \iota_{m\sigma} = \int \left\{1 + x\frac{f'(x)}{f(x)}\right\} f'(x)dx \quad \iota_\sigma = \int \left\{1 + x\frac{f'(x)}{f(x)}\right\}^2 f(x)dx.$$

Writing σ_{0t}, $\partial m_{0t}/\partial\theta_m$ and $\partial\sigma_{0t}/\partial\theta_\sigma$ instead of $\sigma_t(\theta_{0\sigma})$, $\partial m_t(\theta_{0m})/\partial\theta_m$ and $\partial\sigma_t(\theta_{0\sigma})/\partial\theta_\sigma$, we introduce the Fisher information matrices

$$I = \begin{pmatrix} I_\theta & I_{\theta u} \\ I_{u\theta} & I_u \end{pmatrix}, \quad I_\theta = \begin{pmatrix} \iota_m E\frac{1}{\sigma_{0t}^2}\frac{\partial m_{0t}}{\partial\theta_m}\frac{\partial m_{0t}}{\partial\theta_m'} & \iota_{m\sigma} E\frac{1}{\sigma_{0t}^2}\frac{\partial m_{0t}}{\partial\theta_m}\frac{\partial\sigma_{0t}}{\partial\theta_\sigma'} \\ \iota_{m\sigma} E\frac{1}{\sigma_{0t}^2}\frac{\partial\sigma_{0t}}{\partial\theta_\sigma}\frac{\partial m_{0t}}{\partial\theta_m'} & \iota_\sigma E\frac{1}{\sigma_{0t}^2}\frac{\partial\sigma_{0t}}{\partial\theta_\sigma}\frac{\partial\sigma_{0t}}{\partial\theta_\sigma'} \end{pmatrix},$$

$$I_u = E\frac{\partial\log f(U)}{\partial\theta_u}\frac{\partial\log f(U)}{\partial\theta_u'}, \quad I_{\theta u} = I_{u\theta}' = \begin{pmatrix} -E\frac{1}{\sigma_{0t}}\frac{\partial m_{0t}}{\partial\theta_m}\int f'(u)\frac{\partial\log f(u)}{\partial\theta_u'}du \\ -E\frac{1}{\sigma_{0t}^2}\frac{\partial\sigma_{0t}}{\partial\theta_\sigma}\int\left\{u\frac{f'(u)}{f(u)} + 1\right\}\frac{\partial f(u)}{\partial\theta_u'}du \end{pmatrix}.$$

In the sequel, $K > 0$ and $\rho \in [0, 1)$ denote generic constants, or random variables measurable with respect to $\{\boldsymbol{X}_u, u \leq 0\}$, whose values are unimportant and may vary along the text. It can be shown that $\widehat{\vartheta}$ is consistent and asymptotically normal under the following assumptions.

Assumptions A:

(i) the process (X_t) is a non anticipative (in the sense that X_t is a measurable function of $\{\boldsymbol{Y}_{s-1}, U_s, s \leq t\}$) and strictly stationary solution to Model (S1), and when there are exogenous variables in Model (S1), the process $(\boldsymbol{Y}_t, \boldsymbol{U}_t)_t$ is strictly stationary and ergodic;

(ii) Θ is a compact set, whose interior contains ϑ_0;

(iii) $E|X_t|^\tau < \infty$, $E \sup_{\theta_m \in \Theta_m} |m_t(\theta_m)|^\tau < \infty$ and $E \sup_{\theta_\sigma \in \Theta_\sigma} \sigma_t^\tau(\theta_\sigma) < \infty$ for some $\tau > 0$;

(iv) $\sup_{\theta_m \in \Theta_m} |m_t(\theta_m) - \widetilde{m}_t(\theta_m)| \leq K\rho^t$ and $\sup_{\theta_\sigma \in \Theta_\sigma} |\sigma_t(\theta_\sigma) - \widetilde{\sigma}_t(\theta_\sigma)| \leq K\rho^t$;

(v) $\underline{\sigma} := \inf_{\theta_\sigma \in \Theta_\sigma} \sigma_t(\theta_\sigma) > 0$;

(vi) the functions $\theta_m \mapsto m_t(\theta_m)$ and $\theta_\sigma \mapsto \sigma_t(\theta_\sigma)$ admit continuous third order derivatives;

(vii) if $\theta_m, \theta_{0m} \in \Theta_m$, $\theta_m \neq \theta_{0m}$ then $m_t(\theta_m) \neq m_t(\theta_{0m})$ and if $\theta_\sigma, \theta_{0\sigma} \in \Theta_\sigma$, $\theta_\sigma \neq \theta_{0\sigma}$ then $\sigma_t(\theta_\sigma) \neq \sigma_t(\theta_{0\sigma})$ with positive probability;

(viii)

$$\sup_{\theta_m \in \Theta_m} \left\| \frac{\partial m_t(\theta_m)}{\partial \theta_m} - \frac{\partial \widetilde{m}_t(\theta_m)}{\partial \theta_m} \right\| \leq K\rho^t \text{ and } \sup_{\theta_\sigma \in \Theta_\sigma} \left\| \frac{\partial \sigma_t(\theta_\sigma)}{\partial \theta_\sigma} - \frac{\partial \widetilde{\sigma}_t(\theta_\sigma)}{\partial \theta_\sigma} \right\| \leq K\rho^t;$$

(ix) we have

$$E \sup_{\theta_m \in \Theta_m} \left\| \frac{\partial m_t(\theta_m)}{\partial \theta_m} \right\|^\tau < \infty \text{ and } E \sup_{\theta_\sigma \in \Theta_\sigma} \left\| \frac{\partial \sigma_t(\theta_\sigma)}{\partial \theta_\sigma} \right\|^\tau < \infty$$

for some $\tau > 0$;

(x) if $\lambda \in \mathbb{R}^{s_m}$ is non null then $P(\lambda' \partial m_t(\theta_{0m})/\partial \theta_m = 0) < 1$, and if $\lambda \in \mathbb{R}^{s_\sigma}$ is non null then $P(\lambda' \partial \sigma_t(\theta_{0\sigma})/\partial \theta_\sigma = 0) < 1$;

(xi) if $\theta_u, \theta_{0u} \in \Theta_u$, $\theta_u \neq \theta_{0u}$ then $f_{\theta_u} \neq f_{\theta_{0u}}$,

(xii) the information matrix I exists.

The following Lemma 1 summarizes the properties of ϑ defined by (S2). Similar results can be found in the literature (see *e.g.* Pötscher and Prucha [1]). Hence, we do not provide the full proof here, which is available upon request.

Lemma 1 *If Assumptions A holds true, then the MLE $\widehat{\vartheta}$ is consistent. If in addition the matrix I is invertible, then $\sqrt{n}\left(\widehat{\vartheta} - \vartheta_0\right)$ is asymptotically $\mathcal{N}(0, I^{-1})$-distributed.*

Remark Note that when $\alpha_0 = 0$ we have $\partial f_{\theta_{0u}}(x)/\partial \nu = 0$. Therefore $I = I_{\vartheta_0}$ is singular when $\alpha_0 = 0$ or $\beta_0 = 0$. Moreover, the identifiability condition (xi) of Assumptions A is not satisfied in this case, so that the consistency of $\widehat{\vartheta}$ is even not guaranteed when $\alpha_0\beta_0 = 0$.

S2. Proposition 1

Define the vector of the first $s = s_m + s_\sigma + 2$ components of $\vartheta \in \Theta$ as $\underline{\vartheta} = (\theta', \alpha, \beta)'$. Define also the H_0-constrained parameters $\underline{\vartheta}^c = (\theta', 0, 0)'$ and $\underline{\vartheta}_0^c = (\theta_0', 0, 0)'$, and the constrained estimator $\widehat{\underline{\vartheta}}^c = (\widehat{\theta}', 0, 0)'$. For all $\pi \in \Pi$, let $\theta_u(\pi) = (0, 0, \pi')'$, $\vartheta(\pi) = (\theta', \theta_u(\pi)')'$, $\widehat{\vartheta}(\pi) = (\widehat{\theta}', \theta_u(\pi)')'$ and $\vartheta_0(\pi) = (\theta_0', \theta_u(\pi)')'$. Let

$$g_\pi(x) = (g_{\alpha,\pi}(x), g_{\beta,\pi}(x))' \tag{S3}$$

where

$$g_{\alpha,\pi}(x) = (x^2 - 1)\varrho\Phi\left(\frac{\varrho x - \nu}{\sqrt{1 - \varrho^2}}\right) + \phi\left(\frac{\varrho x - \nu}{\sqrt{1 - \varrho^2}}\right)\left\{\frac{(1 - \varrho^2)x - \nu\varrho}{\sqrt{1 - \varrho^2}}\right\},$$
$$g_{\beta,\pi}(x) = (x^2 - 1)\varrho\Phi\left(\frac{-\varrho x + \kappa}{\sqrt{1 - \varrho^2}}\right) + \phi\left(\frac{\varrho x - \kappa}{\sqrt{1 - \varrho^2}}\right)\left\{\frac{\kappa\varrho - (1 - \varrho^2)x}{\sqrt{1 - \varrho^2}}\right\}.$$

Finally, denote by $\underline{\theta}_u = (\alpha, \beta)'$ the first two elements of $\theta_u \in \Theta_u$. The convergence in distribution is denoted by $\xrightarrow{d}$.

Proposition 1 *Assume that the conditions (i)-(viii) of Assumptions A are satisfied. We then have the strong consistency of $\widehat{\theta}$ to θ_0 as $n \to \infty$. Moreover, for all $\pi \in \Pi$, we have under H_0*

$$\sqrt{n}\frac{\partial \widetilde{Q}_n\left\{\vartheta_0(\pi)\right\}}{\partial \underline{\vartheta}} \xrightarrow{d} \mathcal{N}\left(0, I_\pi\right) \text{ as } n \to \infty, \tag{S4}$$

where $\widetilde{Q}_n(\cdot)$ is defined in (S2)*, and the $s \times s$ matrix*

$$I_\pi \text{ is invertible when } \varrho_0 \neq 0 \text{ and } (\nu_0, \kappa_0) \neq (0,0) \tag{S5}$$

and can be consistently estimated by

$$\widehat{I}_\pi = \begin{pmatrix} \widehat{I}_\theta & \widehat{I}_{\theta,\underline{\theta}_u} \\ \widehat{I}_{\underline{\theta}_u,\theta} & I_{\underline{\theta}_u} \end{pmatrix}, \quad \widehat{I}_\theta = \begin{pmatrix} \widehat{I}_m & 0 \\ 0 & \widehat{I}_\sigma \end{pmatrix}, \quad \widehat{I}_{\underline{\theta}_u,\theta} = \widehat{I}'_{\theta,\underline{\theta}_u} = \left(\widehat{I}_{\underline{\theta}_u,m}, \widehat{I}_{\underline{\theta}_u,\sigma}\right) \tag{S6}$$

where

$$\begin{aligned}
\widehat{I}_m &= \frac{1}{n}\sum_{t=1}^n \widehat{D}_{m,t}\widehat{D}'_{m,t}, \qquad \widehat{D}_{m,t} = \frac{1}{\widetilde{\sigma}_t(\widehat{\theta}_\sigma)}\frac{\partial \widetilde{m}_t(\widehat{\theta}_m)}{\partial \theta_m}, \\
\widehat{I}_\sigma &= \frac{2}{n}\sum_{t=1}^n \widehat{D}_{\sigma,t}\widehat{D}'_{\sigma,t}, \qquad \widehat{D}_{\sigma,t} = \frac{1}{\widetilde{\sigma}_t(\widehat{\theta}_\sigma)}\frac{\partial \widetilde{\sigma}_t(\widehat{\theta}_\sigma)}{\partial \theta_\sigma} \\
\widehat{I}_{\underline{\theta}_u,m} &= \boldsymbol{\phi}_\pi \frac{1}{n}\sum_{t=1}^n \widehat{D}'_{m,t}, \quad \widehat{I}_{\underline{\theta}_u,\sigma} = \boldsymbol{\lambda}_\pi \frac{1}{n}\sum_{t=1}^n \widehat{D}'_{\sigma,t},
\end{aligned}$$

with $\boldsymbol{\phi}'_\pi = (\phi(\nu), -\phi(\kappa))$, $\boldsymbol{\lambda}_\pi = E(U_t^2 - 1)g_\pi(U_t)$, $I_{\underline{\theta}_u} = Eg_\pi(U_t)g'_\pi(U_t)$ and $g_\pi(\cdot)$ defined by (S3)*.*

Proof. As conditional asymptotic normality of the Gaussian quasi maximum likelihood estimator (QMLE) has been extensively studied in the literature, we therefore only give detailed proof of (S4) and (S5). Similar and more general results can also be found in the literature (see *e.g.* Amendola and Francq [2]) To lighten the notation, write respectively m_t, $\widetilde{m}_t$, σ_t and $\widetilde{\sigma}_t$ instead of $m_t(\theta_m)$, $\widetilde{m}_t(\theta_m)$, $\sigma_t(\theta_\sigma)$ and $\widetilde{\sigma}_t(\theta_\sigma)$. We have

$$\frac{\partial Q_n\left\{\vartheta(\pi)\right\}}{\partial \theta_m} = \frac{1}{n}\sum_{t=1}^n \left(\frac{X_t - m_t}{\sigma_t}\right)\frac{1}{\sigma_t}\frac{\partial m_t}{\partial \theta_m}, \tag{S7}$$

$$\frac{\partial Q_n\left\{\vartheta(\pi)\right\}}{\partial \theta_\sigma} = \frac{1}{n}\sum_{t=1}^n \left\{\left(\frac{X_t - m_t}{\sigma_t}\right)^2 - 1\right\}\frac{1}{\sigma_t}\frac{\partial \sigma_t}{\partial \theta_\sigma}. \tag{S8}$$

Now note that

$$\Delta_t := \frac{X_t - m_t}{\sigma_t} - \frac{X_t - \widetilde{m}_t}{\widetilde{\sigma}_t} = \frac{X_t(\widetilde{\sigma}_t - \sigma_t) + (\widetilde{m}_t - m_t)\sigma_t + m_t(\sigma_t - \widetilde{\sigma}_t)}{\sigma_t\widetilde{\sigma}_t}.$$

By (iv) and (v) we thus have $|\Delta_t| \leq K\rho^t\left\{|X_t| + \sigma_t + |m_t|\right\}$. Since a relation similar to (S7) holds true for $\partial\widetilde{Q}_n\left\{\vartheta(\pi)\right\}/\partial\theta_m$ we obtain

$$\begin{aligned}
&\left|\frac{\partial Q_n\left\{\vartheta(\pi)\right\}}{\partial \theta_m} - \frac{\partial \widetilde{Q}_n\left\{\vartheta(\pi)\right\}}{\partial \theta_m}\right| \\
&\leq \frac{1}{n}\sum_{t=1}^n |\Delta_t|\frac{1}{\sigma_t}\left|\frac{\partial m_t}{\partial \theta_m}\right| + \left|\frac{X_t - \widetilde{m}_t}{\widetilde{\sigma}_t}\right|\frac{|\widetilde{\sigma}_t - \sigma_t|}{\sigma_t}\left|\frac{\partial m_t}{\partial \theta_m}\right| + \left|\frac{X_t - \widetilde{m}_t}{\widetilde{\sigma}_t}\right|\frac{1}{\widetilde{\sigma}_t}\left|\frac{\partial m_t}{\partial \theta_m} - \frac{\partial \widetilde{m}_t}{\partial \theta_m}\right| \\
&\leq \frac{K}{n}\sum_{t=1}^n \rho^t S_t, \qquad S_t = |X_t| + |m_t| + \sigma_t + \left|\frac{\partial m_t}{\partial \theta_m}\right| + K\rho^t.
\end{aligned}$$

In view of (iii) and (ix) we have $ES_t^s < \infty$. Assuming $s < 1$, we thus obtain

$$E\left(\sum_{t=1}^{\infty} \rho^t S_t\right)^s \leq \sum_{t=1}^{\infty} \rho^{ts} E S_t^s < \infty$$

and thus $\sum_{t=1}^{\infty} \rho^t S_t$ is finite almost surely. Doing similar expansions with (S8), it follows that

$$\sup_{\vartheta\in\Theta} \left\| \frac{\partial Q_n\left\{\vartheta(\pi)\right\}}{\partial\theta} - \frac{\partial \widetilde{Q}_n\left\{\vartheta(\pi)\right\}}{\partial\theta} \right\| = O_P\left(n^{-1}\right). \tag{S9}$$

Note that

$$\begin{aligned}
\frac{\partial f_{\theta_u}(x)}{\partial\alpha} &= -\frac{1}{A_\alpha^{3/2}}(\varrho+\alpha)\phi\left(\frac{x}{\sqrt{A_\alpha}}\right)\Phi\left(\frac{B_\alpha x - \nu A_\alpha}{\sqrt{A_\alpha(1-\varrho^2)}}\right) \\
&\quad - \frac{1}{\sqrt{A_\alpha}}\phi'\left(\frac{x}{\sqrt{A_\alpha}}\right)\frac{x}{A_\alpha^{3/2}}(\varrho+\alpha)\Phi\left(\frac{B_\alpha x - \nu A_\alpha}{\sqrt{A_\alpha(1-\varrho^2)}}\right) \\
&\quad + \frac{1}{\sqrt{A_\alpha}}\phi\left(\frac{x}{\sqrt{A_\alpha}}\right)\Phi'\left(\frac{B_\alpha x - \nu A_\alpha}{\sqrt{A_\alpha(1-\varrho^2)}}\right)\left\{\frac{x - 2\nu(\varrho+\alpha)}{\sqrt{A_\alpha(1-\varrho^2)}} - \frac{(B_\alpha x - \nu A_\alpha)(\varrho+\alpha)}{A_\alpha^{3/2}\sqrt{1-\varrho^2}}\right\},
\end{aligned}$$

where $A_a = 1 + 2a\varrho + a^2$ and $B_a = a + \varrho$.

Therefore, at any $\theta_u = (0, 0, \pi')'$, we have

$$\frac{\partial f_{\theta_u}(x)}{\partial\alpha} = \phi(x)\, g_{\alpha,\pi}(x), \qquad \frac{\partial f_{\theta_u}(x)}{\partial\beta} = \phi(x)\, g_{\beta,\pi}(x). \tag{S10}$$

We then obtain

$$\begin{aligned}
\frac{\partial Q_n\left\{\vartheta(\pi)\right\}}{\partial\underline{\theta}_u} &= \frac{1}{n}\sum_{t=1}^{n} \frac{1}{\phi\left(\frac{X_t - m_t}{\sigma_t}\right)} \frac{\partial}{\partial\underline{\theta}_u} f_{\theta_u}\left(\frac{X_t - m_t}{\sigma_t}\right) \\
&= \frac{1}{n}\sum_{t=1}^{n} g_\pi\left(\frac{X_t - m_t}{\sigma_t}\right).
\end{aligned} \tag{S11}$$

The same equality holds true when Q_n, m_t and σ_t are replaced by $\widetilde{Q}_n$, $\widetilde{m}_t$ and $\widetilde{\sigma}_t$. Using the fact that ϕ and Φ are Lipschitz continuous functions, we have

$$\left\| g_\pi\left(\frac{X_t - m_t}{\sigma_t}\right) - g_\pi\left(\frac{X_t - \widetilde{m}_t}{\widetilde{\sigma}_t}\right) \right\| \leq K|\Delta_t|\left\{X_t^2 + m_t^2 + 1\right\}.$$

By already used arguments, this entails

$$\sup_{\vartheta\in\Theta} \left\| \frac{\partial Q_n\left\{\vartheta(\pi)\right\}}{\partial\underline{\theta}_u} - \frac{\partial \widetilde{Q}_n\left\{\vartheta(\pi)\right\}}{\partial\underline{\theta}_u} \right\| = O_P\left(n^{-1}\right).$$

In view of (S9), we have shown that

$$\sup_{\vartheta\in\Theta} \left\| \frac{\partial Q_n\left\{\vartheta(\pi)\right\}}{\partial\underline{\vartheta}} - \frac{\partial \widetilde{Q}_n\left\{\vartheta(\pi)\right\}}{\partial\underline{\vartheta}} \right\| = O_P\left(n^{-1}\right). \tag{S12}$$

To show (S4), it thus suffices to prove

$$\sqrt{n}\frac{\partial Q_n\left\{\vartheta_0(\pi)\right\}}{\partial\underline{\vartheta}} \xrightarrow{d} \mathcal{N}\left(0, I_\pi\right) \text{ as } n \to \infty. \tag{S13}$$

First note that

$$\sqrt{n}\frac{\partial Q_n(\vartheta_0)}{\partial \underline{\vartheta}} = \frac{1}{\sqrt{n}}\sum_{t=1}^{n}\begin{pmatrix} -\frac{f'_{\theta_{0u}}(U_t)}{f_{\theta_{0u}}(U_t)}\frac{1}{\sigma_{0t}}\frac{\partial m_{0t}}{\partial \theta_m} \\ -\left\{U_t\frac{f'_{\theta_{0u}}(U_t)}{f_{\theta_{0u}}(U_t)}+1\right\}\frac{1}{\sigma_{0t}}\frac{\partial \sigma_{0t}}{\partial \theta_\sigma} \\ \frac{1}{f_{\theta_{0u}}(U_t)}\frac{\partial}{\partial \underline{\theta}_u}f_{\theta_{0u}}(U_t)\end{pmatrix}.$$

where the random variables U_t are independent and $\mathcal{N}(0,1)$-distributed, since under H_0 we have $f_{\theta_{0u}} = \phi$. Using (S10) and (S11), the central limit theorem for martingale differences then entails

$$\sqrt{n}\frac{\partial Q_n\{\vartheta_0(\pi)\}}{\partial \underline{\vartheta}} = \frac{1}{\sqrt{n}}\sum_{t=1}^{n}\begin{pmatrix} U_t\frac{1}{\sigma_{0t}}\frac{\partial m_{0t}}{\partial \theta_m} \\ \left(U_t^2-1\right)\frac{1}{\sigma_{0t}}\frac{\partial \sigma_{0t}}{\partial \theta_\sigma} \\ g_\pi(U_t)\end{pmatrix} \xrightarrow{d} W = \begin{pmatrix} W_m \\ W_\sigma \\ W_{\underline{\theta}_u}\end{pmatrix} \tag{S14}$$

where W is normally distributed with mean 0 and variance

$$I_\pi = \begin{pmatrix} E\frac{1}{\sigma_{0t}^2}\frac{\partial m_{0t}}{\partial \theta_m}\frac{\partial m_{0t}}{\partial \theta'_m} & 0 & E\frac{1}{\sigma_{0t}}\frac{\partial m_{0t}}{\partial \theta_m}EU_tg'_\pi(U_t) \\ 0 & 2E\frac{1}{\sigma_{0t}^2}\frac{\partial \sigma_{0t}}{\partial \theta_\sigma}\frac{\partial \sigma_{0t}}{\partial \theta'_\sigma} & E\frac{1}{\sigma_{0t}}\frac{\partial \sigma_{0t}}{\partial \theta_\sigma}E(U_t^2-1)g'_\pi(U_t) \\ EU_tg_\pi(U_t)E\frac{1}{\sigma_{0t}}\frac{\partial m_{0t}}{\partial \theta'_m} & E(U_t^2-1)g_\pi(U_t)E\frac{1}{\sigma_{0t}}\frac{\partial \sigma_{0t}}{\partial \theta'_\sigma} & Eg_\pi(U_t)g'_\pi(U_t)\end{pmatrix}.$$

Therefore (S13), and thus (S4), are shown. Tedious computations show that $EU_tg_\pi(U_t) = \boldsymbol{\phi}_\pi$. It has not been possible to obtain such an explicit form for

$$\boldsymbol{\lambda}_\pi = E(U_t^2-1)g_\pi(U_t) \quad \text{and} \quad I_{\underline{\theta}_u} = Eg_\pi(U_t)g'_\pi(U_t),$$

but the elements of these matrices can be evaluated by numerical integrations.

We now show by contradiction the invertibility of I_π. If I_π is singular, then there exists a non zero vector $\lambda = (\lambda'_1, \lambda'_2, \lambda_3, \lambda_4)'$, with $\lambda_1 \in \mathbb{R}^{s_m}$, $\lambda_2 \in \mathbb{R}^{s_\sigma}$ and $(\lambda_3, \lambda_4)' \in \mathbb{R}^2$ such that

$$U_t\frac{\lambda'_1}{\sigma_{0t}}\frac{\partial m_{0t}}{\partial \theta_m} + \left(U_t^2-1\right)\frac{\lambda'_2}{\sigma_{0t}}\frac{\partial \sigma_{0t}}{\partial \theta_\sigma} + \lambda_3 g_{\alpha,\pi}(U_t) + \lambda_4 g_{\beta,\pi}(U_t) = 0 \quad \text{a.s.} \tag{S15}$$

Now note that, if $\varrho > 0$ then

$$\lambda_3 g_{\alpha,\pi}(x) + \lambda_4 g_{\beta,\pi}(x) \sim \lambda_3\varrho(x^2-1) \text{ as } x \to +\infty$$

and

$$\lambda_3 g_{\alpha,\pi}(x) + \lambda_4 g_{\beta,\pi}(x) \sim \lambda_4\varrho(x^2-1) \text{ as } x \to -\infty.$$

The equivalences are reversed when $\varrho < 0$. Conditioning on the sigma-field generated by $\{\boldsymbol{X}_u, u < t\}$, (S15) thus entails that, almost surely,

$$\lambda_3\varrho + \frac{\lambda'_2}{\sigma_{0t}}\frac{\partial \sigma_{0t}}{\partial \theta_\sigma} = 0 \quad \text{and} \quad \lambda_4\varrho + \frac{\lambda'_2}{\sigma_{0t}}\frac{\partial \sigma_{0t}}{\partial \theta_\sigma} = 0.$$

The first two equalities entail $\lambda_3 = \lambda_4$, and (S15) thus reduces to

$$U_t\frac{\lambda'_1}{\sigma_{0t}}\frac{\partial m_{0t}}{\partial \theta_m} + \left(U_t^2-1\right)\frac{\lambda'_2}{\sigma_{0t}}\frac{\partial \sigma_{0t}}{\partial \theta_\sigma} + \lambda_3\left\{g_{\alpha,\pi}(U_t) + g_{\beta,\pi}(U_t)\right\} = 0 \quad \text{a.s.} \tag{S16}$$

Note that $x \mapsto g_{\alpha,\pi}(x) + g_{\beta,\pi}(x)$ is linearly independent of $x \mapsto x$ and $x \mapsto x^2 - 1$ when $\nu \neq \kappa$. Therefore (S16) and the conditions (v) and (x) of Assumption A imply $\lambda = 0$, which completes the proof of (S5). ■

S3. Proof of Proposition 2

Proposition 2 *Under H_0 and the other conditions of Proposition 1, for each $\pi \in \Pi_0$ we have*

$$PELM_{n,\pi} \xrightarrow{d} \chi_2^2 \qquad \textit{as } n \to \infty.$$

Proof. From (S14), we have

$$\sqrt{n}\frac{\partial Q_n\left\{\vartheta_0(\pi)\right\}}{\partial \underline{\vartheta}} \xrightarrow{d} \begin{pmatrix} W_m \\ W_\sigma \\ W_{\underline{\theta}_u} \end{pmatrix} \sim \mathcal{N}\left\{0, I_\pi =: \begin{pmatrix} I_m & 0 & I_{m\underline{\theta}_u} \\ 0 & I_\sigma & I_{\sigma\underline{\theta}_u} \\ I_{\underline{\theta}_u m} & I_{\underline{\theta}_u \sigma} & I_{\underline{\theta}_u} \end{pmatrix}\right\}.$$

As $n \to \infty$, we also have a.s.

$$-\frac{\partial^2 Q_n\left\{\vartheta_0(\pi)\right\}}{\partial\theta\partial\theta'} \to I_\theta := \begin{pmatrix} I_m & 0 \\ 0 & I_\sigma \end{pmatrix} \quad \text{and} \quad -\frac{\partial^2 Q_n\left\{\vartheta_0(\pi)\right\}}{\partial\underline{\theta}_u\partial\theta'} \to I_{\underline{\theta}_u\theta} := \begin{pmatrix} I_{\underline{\theta}_u m} & I_{\underline{\theta}_u\sigma} \end{pmatrix}.$$

It is well known that the Gaussian QMLE satisfies

$$\sqrt{n}\left(\widehat{\theta} - \theta_0\right) = I_\theta^{-1}\sqrt{n}\frac{\partial Q_n\left\{\vartheta_0(\pi)\right\}}{\partial\theta} + o_P(1).$$

A Taylor expansion of the function $\widehat{\theta} \mapsto \partial Q_n\left\{\widehat{\vartheta}(\pi)\right\}/\partial\underline{\theta}_u$ around θ_0 and (S12) then yields

$$\begin{aligned} \sqrt{n}\frac{\partial}{\partial\underline{\theta}_u}\widetilde{Q}_n\left\{\widehat{\vartheta}(\pi)\right\} &= \sqrt{n}\frac{\partial}{\partial\underline{\theta}_u}Q_n\left\{\vartheta_0(\pi)\right\} - I_{\underline{\theta}_u,\theta}\sqrt{n}\left(\widehat{\theta} - \theta_0\right) + o_P(1) \\ &\xrightarrow{d} W_{\underline{\theta}_u} - I_{\underline{\theta}_u,\theta}I_\theta^{-1}W_\theta \sim \mathcal{N}\left(0, I_{\underline{\theta}_u} - I_{\underline{\theta}_u,\theta}I_\theta^{-1}I_{\theta\underline{\theta}_u}\right), \end{aligned}$$

using the notation $W_\theta = (W_m', W_\sigma')'$. Noting that $\left\{I_{\underline{\theta}_u} - I_{\underline{\theta}_u,\theta}I_\theta^{-1}I_{\theta\underline{\theta}_u}\right\}^{-1}$ is the lower-right block of I_π^{-1}, the conclusion follows. ■

S4. Properties of PELM$_n$ test statistic and Proposition 3

Following Hansen [3], the supremum of the PELM$_{n,\pi}$ test statistic in (9) is of the form

$$T_n = g\left(\left\{\text{PELM}_{n,\pi}, \pi \in \Pi_0\right\}\right),$$

where $g(\cdot)$ is a continuous mapping from the set of the functionals from Π_0 to $\mathbb{R}$, equipped with the supremum norm. Let the covariance kernel $K(\pi_1, \pi_2) = Es_1(\pi_1)s_1'(\pi_2)$ where

$$s_t'(\pi) = \left(U_t\frac{1}{\sigma_{0t}}\frac{\partial m_{0t}}{\partial\theta_m'}, \left(U_t^2 - 1\right)\frac{1}{\sigma_{0t}}\frac{\partial\sigma_{0t}}{\partial\theta_\sigma'}, g_\pi'(U_t)\right), \quad U_t \sim \mathcal{N}(0,1),$$

where g_π is defined in (S3). Denote by Id_k the identity matrix of size k. Let the $2 \times s$ matrix

$$M_\pi = \left\{I_{\underline{\theta}_u} - I_{\underline{\theta}_u m}I_m^{-1}I_{m\underline{\theta}_u} - I_{\underline{\theta}_u\sigma}I_\sigma^{-1}I_{\sigma\underline{\theta}_u}\right\}^{-1/2}\left(-I_{\underline{\theta}_u m}I_m^{-1}, -I_{\underline{\theta}_u\sigma}I_\sigma^{-1}, Id_2\right)$$

and the covariance kernel $K^*(\pi_1, \pi_2) = M_\pi K(\pi_1, \pi_2)M_\pi'$. Note that $K(\pi, \pi) = I_\pi$ and $K^*(\pi, \pi) = Id_2$. Therefore $\|s_t^*(\pi)\|^2 = s_t^{*'}(\pi)s_t^*(\pi) \sim \chi_2^2$ when $s_t^*(\pi) = M_\pi s_t(\pi)$.

Proposition 3 *Under H_0 and the other conditions of Proposition 1, as $n \to \infty$ we have*

$$PELM_n \xrightarrow{d} PELM_{lim} = \sup_{\pi\in\Pi_0} PELM_\pi \quad \text{and} \quad T_n \xrightarrow{d} g\left(\left\{PELM_\pi, \pi \in \Pi_0\right\}\right),$$

where $PELM_\pi = \|s^*(\pi)\|^2$ and $\{s^*(\pi), \pi \in \Pi_0\}$ denotes a bivariate Gaussian random field on Π_0 with mean zero and covariance kernel $K^*(\cdot,\cdot)$.

One can approximate the distribution of PELM$_{lim}$ by that of

$$\widehat{\text{PELM}}_{lim} = \sup_{\pi\in\Pi_0}\widehat{\text{PELM}}_\pi, \quad \widehat{\text{PELM}}_\pi = \left\|\widehat{M}_\pi\frac{1}{\sqrt{n}}\sum_{t=1}^n\begin{pmatrix}\epsilon_t\widehat{D}_{m,t} \\ \left(\epsilon_t^2 - 1\right)\widehat{D}_{\sigma,t} \\ g_\pi(\epsilon_t)\end{pmatrix}\right\|^2,$$

where $\epsilon_1, \ldots, \epsilon_n$ are independent and $\mathcal{N}(0,1)$ distributed, independently of $\boldsymbol{X}_1, \ldots, \boldsymbol{X}_n$, and where

$$\widehat{M}_\pi = \left(\widehat{I}_{\underline{\theta}_u} - \widehat{I}_{\underline{\theta}_u,\theta}\widehat{I}_\theta^{-1}\widehat{I}_{\theta\underline{\theta}_u}\right)^{-1/2}\left(\widehat{I}_{\underline{\theta}_u,\theta}\widehat{I}_\theta^{-1}, Id_2\right).$$

For $\underline{\alpha} \in (0,1)$, let $c_{\underline{\alpha},n}$ be the $(1-\underline{\alpha})$-quantile of $\widehat{\mathrm{PELM}_{lim}}$ (which can be obtained by Monte Carlo simulations). The supremum PELM test rejects H_0 at the asymptotic level $\underline{\alpha}$ when $\left\{\mathrm{PELM}_n > c_{\underline{\alpha},n}\right\}$.

Proof of Proposition 3. First note that $\mathrm{PELM}_{n,\pi} = \|\widehat{S}_n(\pi)\|^2$ with

$$\widehat{S}_n(\pi) = \left(\widehat{I}^{\underline{\theta}_u}\right)^{1/2}\sqrt{n}\frac{\partial\widetilde{Q}_n\left\{\widehat{\vartheta}(\pi)\right\}}{\partial\underline{\theta}_u}.$$

By the proof of Proposition 2 and (S14), we have

$$\sqrt{n}\frac{\partial}{\partial\underline{\theta}_u}\widetilde{Q}_n\left\{\widehat{\vartheta}(\pi)\right\} = \left(-I_{\underline{\theta}_u\theta}I_\theta^{-1}, Id_2\right)\frac{1}{\sqrt{n}}\sum_{t=1}^n s_t(\pi) + o_P(1).$$

It follows that, as $n \to \infty$,

$$\sup_{\pi\in\Pi_0}\left\|\widehat{S}_n(\pi) - S_n(\pi)\right\| = o_P(1) \quad \text{where} \quad S_n(\pi) = M_\pi\frac{1}{\sqrt{n}}\sum_{t=1}^n s_t(\pi).$$

By Slutsky's lemma we thus have

$$\{\widehat{S}_n(\pi), \pi \in \Pi_0\} \Rightarrow \{s^*(\pi), \pi \in \Pi_0\} \quad \text{iff} \quad \{S_n(\pi), \pi \in \Pi_0\} \Rightarrow \{s^*(\pi), \pi \in \Pi_0\},$$

where $\Rightarrow$ denotes the weak convergence of stochastic processes indexed by $\pi \in \Pi_0$, with continuous trajectories valued in $\mathbb{R}^2$, endowed with the supremum norm.

By the central limit theorem (CLT) of Billingsley [4] for ergodic, stationary and square integrable martingale differences, and the Cramér-Wold device, the finite dimensional distributions of $\{S_n(\pi), \pi \in \Pi_0\}$ converge to that of $\{s^*(\pi), \pi \in \Pi_0\}$. To prove the weak convergence, it remains to show the tightness of $\{S_n(\pi), \pi \in \Pi_0\}$.

A vectorial sequence of random elements is tight when the sequences of each of its components are tight. Moreover a set of probability measures on a finite product space is tight if and only if each of the families of marginal measures is tight (see Corollary 7 of Whitt [5]). In view of Theorem 12.3 of Billingsley [4], the desired tightness result thus follows from

$$\begin{aligned}
E\left\|\frac{1}{\sqrt{n}}\sum_{t=1}^n s_t(\pi_1) - \frac{1}{\sqrt{n}}\sum_{t=1}^n s_t(\pi_2)\right\|^2 &= \operatorname{Tr} E\left\{s_1(\pi_1) - s_1(\pi_2)\right\}\left\{s_1(\pi_1) - s_1(\pi_2)\right\}' \\
&= \int \|g_{\pi_1}(u) - g_{\pi_2}(u)\|^2 \phi(u)du \\
&\leq K\|\pi_1 - \pi_2\|^2,
\end{aligned}$$

with

$$K = \int \sup_{\pi\in\Pi_0}\left\|\frac{\partial g_\pi(u)}{\partial\pi'}\right\|^2\phi(u)du < \infty.$$

The conclusion then comes from the continuous mapping theorem. ■

S5. Recursive estimators for ARMAX-GARCHX model

In this section we consider how the testing procedure can be applied for the ARMA-GARCH models. We allow for exogenous variables since it is often of interest to use economic and financial variables to improve the level and variance predictions (see *e.g.* Francq and Thieu [6] and Sucarrat [7] for references on exogenous

variables in volatility models). Let the vectors of the exogenous variables involved in the mean and variance be denoted by $\boldsymbol{Y}_{mt}$ and $\boldsymbol{Y}_{\sigma t}$, respectively. These vectors can share common components. We assume that $\boldsymbol{Y}_{mt} \in \mathbb{R}^{s_{my}}$, $\boldsymbol{Y}_{\sigma t} \in \mathbb{R}^{s_{\sigma y}}$ and that all the components of $\boldsymbol{Y}_{\sigma t}$ are almost surely positive. Let $\boldsymbol{Y}_t$ be the vector containing all the exogenous variables. For simplicity, let us focus on the first-order model, the ARMAX(1,1)-GARCHX(1,1)

$$\begin{cases} X_t = a_0 X_{t-1} + c_0 + \epsilon_t - b_0 \epsilon_{t-1} + \boldsymbol{c}_0' \boldsymbol{Y}_{m\,t-1} \\ \epsilon_t = \sigma_t^*(\theta_{0\sigma}) U_t, \quad \sigma_t^{*2}(\theta_{0\sigma}) = \omega_0 + \gamma_0 \epsilon_{t-1}^2 + \delta_0 \sigma_{t-1}^{*2}(\theta_{0\sigma}) + \boldsymbol{\omega}_0' \boldsymbol{Y}_{\sigma\, t-1}, \end{cases} \tag{S17}$$

with $\theta_{0m} = (a_0, b_0, c_0, \boldsymbol{c}_0')'$ and $\theta_{0\sigma} = (\omega_0, \gamma_0, \delta_0, \boldsymbol{\omega}_0')'$. The results of this section can be extended to higher-order ARMA-GARCH models, at the price of heavier notation. It will convenient to use the notation $\zeta_{0t} = c_0 + \boldsymbol{c}_0' \boldsymbol{Y}_{m\,t-1}$ and $\varpi_{0t} = \omega_0 + \boldsymbol{\omega}_0' \boldsymbol{Y}_{\sigma\, t-1}$. First note that (S17) is not exactly of the form (S1) because

$$\begin{aligned} \sigma_t^{*2}(\theta_{0\sigma}) &= \varpi_{0t} + \gamma_0 \left\{ \sum_{i=0}^{\infty} b_0^i (X_{t-i-1} - a_0 X_{t-i-2} - \zeta_{0t}) \right\}^2 + \delta_0 \sigma_{t-1}^{*2}(\theta_{0\sigma}) \\ &:= \sigma_{\theta_0}^2(\boldsymbol{X}_{t-1}, \boldsymbol{X}_{t-2}, \dots) \end{aligned}$$

with $\theta_0 = (\theta_{0m}', \theta_{0\sigma}')'$. In particular the function $\widetilde{\sigma}_t(\theta) = \sigma_\theta(\boldsymbol{X}_{t-1}, \boldsymbol{X}_{t-2}, \dots \boldsymbol{X}_1, x_0, \dots)$ does not depend only on θ_σ, but also on the mean parameter θ_m via the approximation of the linear innovation $\widetilde{\epsilon}_t(\theta_m)$, which is recursively defined by

$$\widetilde{\epsilon}_t(\theta_m) = X_t - aX_{t-1} - \zeta_t + b\widetilde{\epsilon}_{t-1}(\theta_m), \quad t = 2, \dots$$

for some fixed initial value $\widetilde{\epsilon}_1(\theta_m) = \widetilde{\epsilon}_1$ and obvious notations. It is easy to see that the results of Propositions 1-3 remain valid, provided θ_σ and $\widehat{\theta}_\sigma$ are replaced by θ and $\widehat{\theta}$, with the estimators

$$\widehat{I}_\theta = \frac{1}{n} \sum_{t=1}^{n} \frac{1}{\widetilde{\sigma}_t^2(\widehat{\theta})} \frac{\partial \widetilde{\epsilon}_t(\widehat{\theta}_m)}{\partial \theta} \frac{\partial \widetilde{\epsilon}_t(\widehat{\theta}_m)}{\partial \theta'} + \frac{1}{2} \frac{1}{n} \sum_{t=1}^{n} \frac{1}{\widetilde{\sigma}_t^4(\widehat{\theta})} \frac{\partial \widetilde{\sigma}_t^2(\widehat{\theta})}{\partial \theta} \frac{\partial \widetilde{\sigma}_t^2(\widehat{\theta})}{\partial \theta'} \tag{S18}$$

and

$$\widehat{I}_{\theta, \underline{\theta}_n} = \frac{-1}{n} \sum_{t=1}^{n} \frac{1}{\widetilde{\sigma}_t(\widehat{\theta})} \frac{\partial \widetilde{\epsilon}_t(\widehat{\theta}_m)}{\partial \theta} E U_1 g_\pi'(U_1) + \frac{1}{2n} \sum_{t=1}^{n} \frac{1}{\widetilde{\sigma}_t^2(\widehat{\theta})} \frac{\partial \widetilde{\sigma}_t^2(\widehat{\theta})}{\partial \theta} E(U_1^2 - 1) g_\pi'(U_1). \tag{S19}$$

In the previous matrices the derivatives are computed recursively by

$$\frac{\partial \widetilde{\epsilon}_t(\theta_m)}{\partial \theta} = \begin{pmatrix} -X_{t-1} \\ \widetilde{\epsilon}_{t-1}(\theta_m) \\ -1 \\ -\boldsymbol{Y}_{m\,t-1} \\ 0_{s_\sigma} \end{pmatrix} + b \frac{\partial \widetilde{\epsilon}_{t-1}(\theta_m)}{\partial \theta}, \quad \frac{\partial \widetilde{\sigma}_t^2(\theta)}{\partial \theta} = \begin{pmatrix} 2\gamma \widetilde{\epsilon}_{t-1}(\theta_m) \frac{\partial \widetilde{\epsilon}_{t-1}(\theta_m)}{\partial \theta_m} \\ 1 \\ \widetilde{\epsilon}_{t-1}^2(\theta_m) \\ \widetilde{\sigma}_{t-1}^2(\theta) \\ \boldsymbol{Y}_{\sigma\, t-1} \end{pmatrix} + \delta \frac{\partial \widetilde{\sigma}_{t-1}^2(\theta)}{\partial \theta}. \tag{S20}$$

Note also that the GARCH equation is not written in the standard form (more precisely, the "volatility" $\sigma_t^{*2}(\theta_{0\sigma})$ is not necessarily equal to the conditional variance of X_t) because, in general, $EU_t \neq 0$ and $EU_t^2 \neq 1$ for WSN distributions. It is however easy to see that (i) of Assumptions A is satisfied if

$$|a_0| < 1, \quad |b_0| < 1, \quad E \log(\gamma_0 U_1^2 + \delta_0) < 0 \tag{S21}$$

and the process $(\boldsymbol{Y}_t, \boldsymbol{U}_t)_t$ is strictly stationary and ergodic. The strict stationarity condition also entails the existence of a fractional moment, as required in the first part of (iii) (see Lemma 2.3 in Berkes, Horváth and Kokoszka [8] [8] for standard GARCH models and Lemma 2 in Francq and Thieu [6] when there exist exogenous variables). Assuming that for all $\theta_m = (a, b, c, \boldsymbol{c}')' \in \Theta_m$ and all $\theta_\sigma = (\omega, \gamma, \delta, \boldsymbol{\omega}')' \in \Theta_\sigma$,

$$|a| < 1, \quad |b| < 1, \quad \omega \geq \underline{\omega} > 0, \quad \gamma \geq 0, \quad 0 \leq \delta < 1, \quad \boldsymbol{\omega} \geq 0,$$

where the last inequality is componentwise, the other moment conditions of (iii), as well as the conditions (iv)-(v) and (viii) are always satisfied (see *e.g.* Francq and Zakoïan, [9]; and Francq and Thieu [6]). Condition

(vi) is trivially satisfied. Conditions (viii) and (ix) are also entailed by the previous conditions and the specific form of the conditional moments. When there is no exogenous variables, the identifiability conditions (vii) and (x) hold true when

$$a_0 \neq b_0 \qquad \text{and} \qquad \gamma_0 > 0. \tag{S22}$$

When there are exogenous variables, additional assumptions are required. Note that $\mathcal{F}_t = \sigma\{U_{t-k}, \boldsymbol{Y}_{\sigma\, t-k}, k \geq 0\}$. Let $\mathcal{F}_{t,i} = \sigma\{U_{t-j}, j > i, \boldsymbol{Y}_{t-k}, k > 0\}$. We now require a condition slightly stronger than **A4** in Francq and Thieu [6], by assuming that

$$\text{for all } i \geq 0, \text{ the support of the distribution of } U_{t-i} \text{ given } \mathcal{F}_{t,i} \text{ is the real line} \tag{S23}$$

When there are no covariates, we have $\mathcal{F}_{t,i} = \mathcal{F}_{t-i-1}$ and thus (S23) is satisfied because the support of the WSN distribution is the real line. More generally, (S23) rules out the existence of redundant information between the exogenous variables and the past observations. For example, the assumption precludes that $\boldsymbol{Y}_t = \boldsymbol{Y}_{\sigma\, t} = \epsilon_t^2$, since in this case $\mathcal{F}_{t,1} = \sigma\left(U_{t-1}, U_{t-2}, U_{t-3}, \dots\right)$, and the distribution of $U_{t-1} \mid \mathcal{F}_{t,1}$ is degenerated. This is necessary since the parameters γ_0 and $\boldsymbol{\omega}_0$ of the volatility $\sigma_t^{*2} = \omega_0 + \gamma_0 \varepsilon_{t-1}^2 + \delta_0 \sigma_{t-1}^{*2} + \boldsymbol{\omega}_0' \boldsymbol{Y}_{\sigma\, t-1} x_{t-1}^2$ are clearly not identifiable in that case.

To avoid multicollinearity of the explanatory variables, assume also that

$$\text{if } \boldsymbol{c} \text{ is a non- zero vector of } \mathbb{R}^{s_{my}} \text{ then } \boldsymbol{c}'\boldsymbol{Y}_{m\,1} \text{ is not degenerated} \tag{S24}$$

and

$$\text{if } \boldsymbol{\omega} \text{ is a non- zero vector of } \mathbb{R}^{s_{\sigma y}} \text{ then } \boldsymbol{\omega}'\boldsymbol{Y}_{\sigma\,1} \text{ is not degenerated.} \tag{S25}$$

Remark 3.5 in Francq and Zakoïan [9] shows that the information matrix I may not exist without moment condition on X_t, when the ARMA part is present. Condition (xii) is guaranteed under the moment condition $E\epsilon_t^4 < \infty$, given by

$$E\|\boldsymbol{Y}_t\|^4 < \infty \quad \text{and} \quad 2\gamma_0\delta_0 EU_1^2 + \delta_0^2 + \gamma_0^2 EU_1^4 < 1.$$

Finally, Lemma 2 below shows that under the previous assumptions, the identifiability condition (iv) is satisfied.

Lemma 2 *Under S21, let (X_t) be a stationary ergodic and non anticipative solution of Model S17. Assume* (S23)*,* (S24) *and* (S25)*. If $m_t(\theta_m) = m_t(\theta_{0m})$ a.s. then $\theta_m = \theta_{0m}$. If $\sigma_t(\theta_\sigma) = \sigma_t(\theta_{0\sigma})$ a.s. then $\theta_\sigma = \theta_{0\sigma}$.*

Proof. If $m_t(\theta_m) = m_t(\theta_{0m})$ a.s. then $\epsilon_t(\theta_m) := X_t - m_t(\theta_m) = \epsilon_t$ a.s. Denoting by L the lag operator, we then have

$$\left\{\frac{1-aL}{1-bL} - \frac{1-a_0L}{1-b_0L}\right\} X_t = \left\{\frac{\boldsymbol{c}_0'L}{1-b_0L} - \frac{\boldsymbol{c}'L}{1-bL}\right\} \boldsymbol{Y}_{m\,t} + \left\{\frac{c_0}{1-b_0} - \frac{c}{1-b}\right\} \quad \text{a.s.}$$

In view of the first condition in (S22), if $a \neq a_0$ or $b \neq b_0$ then there exist a sequence $(c_i)_{i\geq 0}$ such as $c_{i_0} \neq 0$, a constant e and a sequence of vectors $(\boldsymbol{d}_i)_{i\geq 1}$ such that

$$\sum_{i=i_0}^{\infty} c_i X_{t-i} + \sum_{i=1}^{\infty} \boldsymbol{d}_i' \boldsymbol{Y}_{m\,t-i} = e.$$

In this case U_{t-i_0} would be a measurable function of $\{U_{t-i}, i > i_0; \boldsymbol{Y}_{t-k}, k > 0\}$, that is U_{t-i_0} would be $\mathcal{F}_{t,i_0}$-measurable, which is impossible under (S23). It is the easy to show that we must have $c = c_0$ and $\boldsymbol{c} = \boldsymbol{c}_0$ under (S24). We thus have shown the first result of the lemma. The second result is shown similarly (see also the proof of Theorem 1 in Francq and Thieu [6]). ■

References

[1] Pötscher, B.M., Prucha, I.R. Dynamic Nonlinear Econometric Models. Springer, Berlin, 1997.

[2] Amendola, A., Francq, C. Concepts of and tools for nonlinear time series modelling. Handbook of Computational Econometrics. Eds: D. Belsley and E. Kontoghiorghes. Wiley, 2009; 377–427. https://doi.org/10.1002/9780470748916.ch10

[3] Hansen, B.E. Inference when a nuisance parameter is not identified under the null hypothesis. Econometrica. 1996; 64, 413–430. https://doi.org/10.2307/2171789

[4] Billingsley, P. Statistical Inference for Markov Processes. University of Chicago Press, 1961.

[5] Whitt, W. Weak convergence of probability measures on the function space $C[0,\infty)$. The Annals of Mathematical Statistics. 1970; 41, 939–944. https://doi.org/10.1214/aoms/1177696970

[6] Francq, C., Thieu, Q. QML inference for volatility models with covariates. Econometric Theory. 2019; 35, 37–72. https://doi:10.1017/S0266466617000512

[7] Sucarrat, G. Garchx: Flexible and robust GARCH-X modeling. R Journal. 2021; 13, 276–291. https://journal.r-project.org/archive/2021/RJ-2021-057/index.html

[8] Berkes, I., Horváth, L., Kokoszka, P. GARCH processes: structure and estimation. Bernoulli. 2003; 9, 201–227. https://doi.org/10.3150/bj/1068128975

[9] Francq, C., Zakoïan, J-M. Maximum likelihood estimation of pure GARCH and ARMA-GARCH processes. Bernoulli. 2004; 10, 605–637. https://doi.org/10.3150/bj/1093265632
