# Supplementary material for: Testing for the footprints of stabilization economic policy in forecast errors
Source: PLoS One. 2025 Dec 1;20(12):e0336495. doi: 10.1371/journal.pone.0336495 (PMC12668634; doi:10.1371/journal.pone.0336495)
Supplement: Supplement 2 — (PDF) [file pone.0336495.s002.pdf]

Testing for the Footprints of Stabilization Economic Policy in Forecast Errors

Wojciech Charemza[1,2], Christian Francq[3], Radu Lupu[4], Svetlana Makarova[*5], and Jean-Michel Zakoïan[3]

[1]Vistula University, Poland

[2]University of Leicester, UK

[3]CREST, Paris, France

[4]Bucharest University of Economic Studies and Institute for Economic Forecasting, Romanian Academy, Romania

[5]University College London, UK

SUPPLEMENT 2

Additional simulation and empirical results

[*] Corresponding author: s.makarova@ucl.ac.uk

Part 1. Optimal number of drawings of ν_0 and κ_0 for the PELM_n test

Table S2.1 shows the results of computing empirical quantiles of the $\mathrm{PELM}_n(\text{narrow})$ and $\mathrm{PELM}_n(\text{wide})$ tests, computed for the ARMA-GARCH model (10) in the main body of the paper. All settings are as in the first part of Section 4 devoted to the analysis of size distortion, that is, under the null hypothesis that $\alpha_0 = \beta_0 = 0$, with the exception that the number of drawings of ν_0 and κ_0 changes from 40 to 140 with a step of 5, which gives 21 sets of results, each for a different number of drawings. For each number of drawings, the number of replications is set at 5000. Next, for a selected quantile of the PELM_n, statistic, the simulated values of this quantile are ordered by the number of drawings, from the smallest (4) to the largest (140). For such a series, three tests for randomness of the series are applied, namely the von Neumann ratio test (VN), ranked von Neumann test (RVN), also known as the Bartels test, and the runs test (RUNS). Descriptions of these tests can be found, for instance, in Brockwell and Davis [1] and Gibbons and Chakraborti [2].

The precise settings for the null and alternative hypotheses are the following:

1. Von Neumann ratio test:
   - Null (H_0): The observations are independent (random).
   - Alternative (H_1): The observations are not independent (non-random), usually showing positive or negative autocorrelation.
2. Ranked von Neumann test (Bartels test):
   - Null (H_0): The sequence of ranks is random (independent).
   - Alternative (H_1): The sequence is not random (has serial dependence, monotone trend, or clustering).
3. Runs test:
   - Null (H_0): The sequence is random (the order of values above/below the median, or of two symbols, is independent).
   - Alternative (H_1): The sequence is not random (too many or too few runs appear, indicating clustering or alternating patterns).

Table S2.1A: Results of the randomness tests
Number of drawings: from 40 to 140

$\mathrm{PELM}_n(\text{narrow})$ statistic

test	statistic	p-value	p-value_b	statistic	p-value	p-value_b
	quantile: 0.95			quantile: 0.99		
VN test	0.713	0.167	0.171	0.672	0.114	0.125
Runs test	-1.111	0.266	0.165	-1.56	0.119	0.195
RVN Test	0.704	0.154	0.165	0.724	0.184	0.195

$\mathrm{PELM}_n(\text{wide})$ statistic

test	statistic	p-value	p-value_b	statistic	p-value	p-value_b
	quantile: 0.95			quantile: 0.99		
VN test	0.713	0.167	0.171	0.672	0.114	0.125
Runs test	-1.111	0.266	0.165	-1.56	0.119	0.195
RVN Test	0.704	0.154	0.165	0.724	0.184	0.195

Legend: statistic: value of the statistic; p-value: analytical (large sample) p-value; p-value_b bootstrapped p-value (No of bootstrap replications: 5,000)

Results in Table S2.1A indicate that, for the 0.95 and 0.99 quantiles and for both PELM_n(narrow) and PELM_n(wide) tests, the null hypothesis of the randomness of the series of quantiles cannot be rejected; that is, an increase in the number of drawings does not add any systematic element to the quantiles' estimates. Consequently, it seems reasonable to use the cheapest, in terms of the computational costs, setting where the number of drawings is set to 40.

Table S2.1B shows the coefficients of variation, that is, standard deviations divided by the means, for the series of simulated quantiles.

Table S2.1B: Coefficients of variation the simulated quantiles

	0.95 quantile	0.99 quantile
PELM_n(narrow)	0.007	0.016
PELM_n(wide)	0.016	0.024

Results in Table S2.1B indicate that the dispersion of simulated quantiles is very small, in the range from 1 to 2.5% of the means. It reinforces the rationality of using the cheapest number of drawings for computing both variants of the PELM_n test.

Part 2. Country codes and summary statistics

Table S2.2. Country codes

Code	Country	Code	Country
AUS	Austria	JAP	Japan
AUL	Australia	KOR	Korea
BEL	Belgium	MEX	Mexico
BRA	Brazil	NET	Netherlands
CAN	Canada	POL	Poland
CHL	Czech Republic	POR	Portugal
CZE	Chile	RUS	Russia
DEN	Denmark	SAF	South Africa
FIN	Finland	SPA	Spain
FRA	France	SVK	Slovakia
GER	Germany	SVN	Slovenia
HUN	Hungary	SWE	Sweden
ICE	Iceland	SWI	Switzerland
IND	India	TUR	Turkey
INZ	Indonesia	UKM	United Kingdom
IRE	Ireland	USA	United States
ITA	Italy		

Table S2.3. Summary statistics for differences
for the period 1 January 2012 – 31 December 2019.

Country	No. obs.	mean	st. dev.	skewness	kurtosis	median
AUS	2086	-0.002	0.037	0.486	6.279	-0.002
AUL	2086	-0.001	0.051	0.105	4.978	0.000
BEL	2086	-0.002	0.039	0.436	8.135	-0.003
BRA	2086	-0.002	0.131	1.026	19.155	0.000
CAN	2086	0.000	0.040	0.139	3.394	0.000
CHL	2086	-0.001	0.045	0.270	41.177	0.000
CZE	2086	-0.001	0.039	0.399	9.462	0.000
DEN	2086	-0.001	0.039	1.104	10.919	-0.001
FIN	2086	-0.001	0.037	0.631	6.523	-0.001
FRA	2086	-0.001	0.038	0.356	6.205	-0.002
GER	2086	-0.001	0.037	0.500	5.133	-0.002
HUN	2086	-0.004	0.083	-0.726	17.129	0.000
ICE	2086	-0.002	0.060	-0.232	13.577	0.000
IND	2086	-0.001	0.052	0.707	20.943	0.000
INZ	2086	0.000	0.074	0.212	8.846	0.000
IRE	2086	-0.004	0.051	-0.763	21.906	-0.004
ITA	2086	-0.003	0.067	0.323	8.424	-0.002
JAP	2086	0.000	0.016	0.546	10.331	0.000
KOR	2086	-0.001	0.032	0.052	6.858	0.000
MEX	2086	0.000	0.067	0.305	9.966	0.000
NET	2086	-0.001	0.036	0.646	5.385	-0.002
POL	2086	-0.002	0.051	0.452	6.592	0.000
POR	2086	-0.006	0.132	1.542	71.636	-0.004
RUS	2086	-0.001	0.140	1.693	186.046	0.000
SAF	2086	0.000	0.074	1.848	28.381	0.000
SPA	2086	-0.002	0.066	-0.023	10.851	-0.003
SVK	2086	-0.002	0.044	-10.474	297.020	0.000
SVN	2086	-0.003	0.079	0.685	35.515	-0.002
SWE	2086	-0.001	0.037	0.934	10.553	-0.001
SWI	2086	-0.001	0.029	0.736	9.756	-0.001
TUR	2086	0.001	0.199	0.654	17.549	0.000
UKM	2086	-0.001	0.045	0.063	4.703	-0.001
USA	2086	0.000	0.043	0.225	4.303	0.000

Part 3. Unit root tests

For all 33 countries during the empirical analysis period and the application of the PELM tests, from January 1, 2012, to December 31, 2019, we performed seven unit root tests to examine the first differences of daily time series of the sovereign bond yields. These tests are: the feasible point optimal test (PT), modified PT (MPT), GLS-detrended ADF, Phillips-Perron Za test, and modified Za (MZA), (see Ng and Perron [3]; Perron and Qu [4]) and MSB and MZT tests (see Carrion-i-Silvestre; Kim and Perron [5]). The null hypothesis is that the process is I(1), that is, stationary in first differences, with the alternative of I(0) (stationarity). In summary, for most tests and most countries, the null of stationarity has been confirmed; therefore, the series are regarded as stationary. The detailed results are given in Tables S2.4 and S2.5 below.

Table S2.4. Unit Root tests results for first differences of yields by country
for the period 1 January 2012 – 31 December 2019

Country	No. of rejections	Frequency	Country	No. of rejections	Frequency
AUS	5	0.71	JAP	7	1.00
AUL	2	0.29	KOR	7	1.00
BEL	6	0.86	MEX	6	0.86
BRA	7	1.00	NET	7	1.00
CAN	7	1.00	POL	7	1.00
CHL	7	1.00	POR	7	1.00
CZE	7	1.00	SAF	7	1.00
DEN	7	1.00	SAF	7	1.00
FIN	7	1.00	SPA	6	0.86
FRA	7	1.00	SVK	7	1.00
GER	7	1.00	SVN	7	1.00
HUN	2	0.29	SWE	7	1.00
ICE	7	1.00	SWI	7	1.00
IND	7	1.00	TUR	7	1.00
INZ	7	1.00	UKM	7	1.00
IRE	7	1.00	USA	6	0.86
ITA	7	1.00			

Legend: No. of rejections: number of rejections, out of seven tests performed, the null hypothesis of I(1). Frequency: frequency of rejection of the null hypothesis.

Table S2.5. Average frequency of rejections of the null of I(1) for unit root tests for first differences of yields by tests for the period 1 January 2012 – 31 December 2019

Test	Average No. of rejections
PT, feasible point optimal	0.79
MPT, PT modified	0.94
ADF, GLS detrended	1.00
Phillips-Perron Za	1.00
MZA, Za modified	0.91
MSB, Silvestre-Kim-Perron	0.94
MZT, Silvestre-Kim-Perron	0.94

Part 4. Results for different fiscal balance indicators and for the Brexit referendum crisis

We computed the paired *t*-test for differences (the 'after treatment effects' test) for four different fiscal balance indicators, namely:

Budget deficit: (BudDef); Revenue minus expenditure (Rev-Exp); Net lending/borrowing (NLB); Primary net lending/borrowing: (PNLB).

Data for all fiscal balance indicators are collected from various sites containing the International Monetary Fund statistics. Together with links to the primary data, they are available in Supplement 3.

Table S2.6 contains a summary of paired *t*-test results by giving the bootstrap p-values for the *t*-paired statistics for groups of countries where the $\mathrm{PELM}_{n,\pi}$ statistics are, respectively, significant at the 0.05 level of significance (Group A), and insignificant (Group B); see Section 5 of the paper for a more detailed description of the selection of these groups. These p-values are denoted as pval(A) and pval(B). The last two columns give ratios of the number of countries in Groups A and B, where there is an improvement in fiscal measures between the aftershock and final years.

Table S2.6 Additional paired test results

	fiscal measure	years		paired test bootstrap pvals		fractions of improvement	
Crisis		aft.shck	final	pval(A)	pval(B)	in A	in B
Brexit	BudDef	2016	2018	0.516	0.791	0.607	0.600
Brexit	Rev-Exp	2016	2017	0.017	0.366	0.778	0.600
Brexit	NLB	2016	2017	0.023	0.224	0.778	0.800
Brexit	PNLB	2016	2017	0.012	0.648	0.667	0.600
Rouble	BudDef	2015	2017	0.002	0.599	0.696	0.600
Rouble	Rev-Exp	2015	2016	0.111	0.483	0.682	0.600
Rouble	NLB	2015	2016	0.039	0.483	0.727	0.600
Rouble	PNLB	2015	2016	0.085	0.018	0.727	0.800

Brief analysis of the results in this table is in the main paper, Section 6.

References

1. Brockwell, P. J., Davis, R. A. Introduction to Time Series and Forecasting (3rd ed.). 2016. Springer.
2. Gibbons, J. D., Chakraborti, S. Nonparametric Statistical Inference (6th ed.). 2021. Chapman & Hall.
3. Ng, S., Perron, P. Lag length selection and the construction of unit root tests with good size and power. Econometrica, 2021; 69(6), 1519-1554. https://doi.org/10.1111/1468-0262.00256

4. Perron, P., Qu, Z. A simple modification to improve the finite sample properties of Ng and Perron's unit root tests. Economics Letters. 2007; 94(1), 12-19. https://doi.org/10.1016/j.econlet.2006.06.009

5. Carrion-i-Silvestre, J.L., Kim, D., Perron, P. 2009. GLS-based unit root tests with multiple structural breaks under both the null and the alternative hypotheses. Econometric Theory. 2009; 25(6), 1754-1792. https://doi.org/10.1017/S0266466609990326
